# Decoding meningioma heterogeneity and neoplastic cell—macrophage interaction through single-cell transcriptome profiling across pathological grades

**DOI:** 10.1186/s12967-023-04445-4

**Published:** 2023-10-25

**Authors:** Hailang Fan, Lairong Song, Jian Fan, Junpeng Ma, Xiaojie Li, Junting Zhang, Jian Hu, Zhen Wu, Dake Zhang, Liang Wang

**Affiliations:** 1 Key Laboratory of Biomechanics and Mechanobiology, Ministry of Education, Beijing Advanced Innovation Center for Biomedical Engineering, School of Engineering Medicine, Beihang University, Beijing, 100191 China; 2 Department of Neurosurgery, Beijing Tiantan Hospital, Capital Medical University, Beijing, 100070 China; 3 Department of Urology, Peking University First Hospital, Institute of Urology, National Urological Cancer Center, Urogenital Diseases (Male) Molecular Diagnosis and Treatment Center, Peking University, Beijing, 100871 China; 4 Department of Cancer Biology, The University of Texas MD Anderson Cancer Center, Houston, TX 77054-1901 USA; 5 MD Anderson Cancer Center UTHealth Graduate School of Biomedical Sciences, Houston, TX 77225-0334 USA

**Keywords:** Meningiomas, scRNA-seq, Heterogeneity, *MIF*, *CD74*

## Abstract

**Background:**

Analyzing meningioma of distinct pathological types at the single-cell level can provide new and valuable insights into the specific biological mechanisms of each cellular subpopulation, as well as their vital interplay within the tumor microenvironment.

**Methods:**

We recruited patients diagnosed with four distinct types of meningioma and performed single-cell RNA sequencing on their tumor samples, concurrently analyzing a publicly available dataset for comparison. Next, we separated the cells into discrete clusters and identified their unique identities. Using pseudotime analysis, we demonstrated cellular differentiation and dynamics. To investigate biological function, we employed weighted gene co-expression network analysis, gene regulatory network, and gene set enrichment analysis. Additionally, we conducted cell–cell communication analyses to characterize interactions among different clusters and validated a crucial interaction using multiple immunofluorescence staining.

**Results:**

The single-cell transcriptomic profiles for five meningioma of different pathological types demonstrated that neoplastic cells exhibited high inter-sample heterogeneity and diverse biological functions featured by metabolic regulation. A small cluster of neoplastic cells (N5 cluster, < 3%) was most proliferative, indicated by high expression of *MKI67* and *TOP2A*. They were primarily observed in our atypical and transitional meningioma samples and located at the beginning of the pseudotime differentiation branch for neoplastic cells. Macrophages, the most abundant immune cells present, showed two distinct developmental trajectories, one promoting and the other suppressing meningioma growth, with the MIF-CD74 interaction serving as the primary signaling pathway for MIF signals in the tumor environment. Unexpectedly, despite its small cluster size, the N5 cluster demonstrated a significant contribution in this interaction. By staining pathological sections of more samples, we found that this interaction was widely present in different types of meningiomas.

**Conclusions:**

Meningioma neoplastic cells' diverse types cause inter-sample heterogeneity and a wide range of functions. Some proliferative neoplastic cell may educate macrophages, which promotes tumorigenesis possibly through the MIF-CD74 interaction. It provides novel clues for future potential therapeutic avenues.

**Supplementary Information:**

The online version contains supplementary material available at 10.1186/s12967-023-04445-4.

## Background

Meningioma, a type of neoplasm that originates from the meninges of the central nervous system, constitutes the most frequent primary intracranial tumor of the CNS [1]. Meningiomas present a high degree of heterogeneity, with an annual incidence of 7.86 per 100,000 and accounting for 37% of all primary intracranial tumor [2]. The latest 2021 WHO brain tumor classification lists fifteen meningioma subtypes, classifying them based on their histological and biological features [3]. Meningiomas demonstrate significant clinical heterogeneity regarding disease symptoms, tissue pathology, recurrence rates, clinical invasiveness, and outcomes. Moreover, meningiomas can be categorized based on their molecular properties, and different subgroups manifest distinct clinical outcomes and recurrence rates [4, 5].

Single-cell RNA sequencing (scRNA-seq) represents an effective method to unravel heterogeneity, classify cell subpopulations, and identify cellular origins in human cancers. In addition, scRNA-seq can identify shared features among different individuals with the same disease, thereby enhancing our comprehension of common mechanisms underlying the disease and potentially providing leads for discovering novel therapeutic targets. In previous single-cell transcriptomic analyses of meningiomas, cell identification indicated that most cells were tumor cells (69%), while 14% were immune cells (macrophages and T cells), 10% were fibroblasts, and 6% were endothelial cells [4]. Notably, single-cell transcriptomics can uncover the significant heterogeneity of meningioma tumor cells, which tend to cluster according to patients. Additionally, immune cells are more abundant in meningiomas compared to normal dura mater tissue, and macrophages present in both may have different origins [6]. By identifying immune-enriched meningiomas based on DNA methylation, single-cell transcriptome analysis has revealed an increased proportion of immune cells in this subtype. Moreover, the expression of *HLA-DRB5*, *HLA-DRB1*, *HLA-DQA1*, *HLA-DMA*, and *HLA-DPB1* was found to be increased [7]. Currently, scRNA-seq studies on meningiomas primarily focus on characterizing the cellular landscape, providing initial insights into the immune landscape. Nevertheless, detailed analysis of the differential expression patterns of distinct cell types, as well as exploration of the interactions between various cell types, remain necessary.

Additional investigations have revealed that macrophages represent the predominant cell type in the tumor microenvironment (TME) of meningiomas and that the proportion of macrophages is greater in WHO grade 2 meningiomas than in WHO grade 1 meningiomas[8, 9]. Macrophages have a complex role in tumors, and in the presence of tumor cells, they can concurrently exhibit two different states: promoting and inhibiting tumor growth [10]. In a particular study, targeting the *CSF-1*/*CSF-1R* signaling pathway to reduce the immunosuppressive function of macrophages has been identified as a promising immunotherapeutic strategy for high-grade meningiomas [11].

In this study, we recruited five meningioma patients with varying pathological types or WHO grades and analyzed their tumor samples at the single-cell level to generate a single-cell transcriptome profile of meningiomas. We conducted further functional characterization to better understand the gene expression differences in individual neoplastic cell heterogeneity. Our investigation also revealed that neoplastic cells may be capable of universally educating macrophages to promote tumor development, potentially through MIF-CD74 interaction. Targeting MIF-CD74 may therefore represent a promising strategy for the development of immunotherapeutic approaches for the treatment of meningioma.

## Methods

### Patients and clinical information

Five patients were recruited by Beijing Tiantan Hospital, Capital Medical University between 2017 and 2018. Based on imaging studies, a diagnosis of meningioma was suggested and subsequently confirmed by postoperative pathology reports. All enrolled patients provided written informed consent, and the study protocols were approved by the ethical review board of Beijing Tiantan Hospital, Capital Medical University (KY2017-022-01). Table 1 displays their comprehensive clinical information.

**Table 1:** Clinical characteristics of meningiomas patients

Sample ID	Sex	Pathology	WHO	Radiotherapy	Surgery time/years	Recurrence
M.p.fib	F	Fibrous	1	N	1	Y
M.r.tra1	F	Transitional	1	Y	3	N
M.r.tra2	F	Transitional	2	Y	4	Y
M.r.aty	M	Atypical	2	Y	2	N
M.r.cle	F	Clear cell	2	N	2	N

### HE staining

Meningioma tissue samples underwent successive perfusion with saline and 4% paraformaldehyde. The tissues were fixed for 24 h using 4% paraformaldehyde, followed by dehydration with 70% ethanol and paraffin embedding. The resulting meningioma samples were then sectioned continuously and subjected to hematoxylin–eosin (HE) staining, following established protocols.

### Sample collection and single-cell suspension

During surgery, the specimens were promptly immersed in chilled RPMI1640 medium after being excised from the whole tumor. The samples were subsequently trimmed with scissors and washed thrice with Hank's Balanced Salt Solution (HBSS) to eliminate blood cells and burnt tissues. Subsequently, the tissue was cut into pieces of less than 2 mm diameter and underwent tissue digestion in a digestive enzyme mixture containing 20 ml of pre-warmed RPMI1640 (ThermoFisher Scientific), 200U/ml type IV collagenase (ThermoFisher Scientific), and 10% Fetal Bovine Serum (FBS, ThermoFisher Scientific) for 40 min at 37 °C while being shaken. Cell suspensions were filtered through a 70 μm filter and centrifuged at 1000 rpm for 5 min. The cells were washed twice, and resuspended in HBSS with 10% FBS. BD FACS lysing solution (BD company) was used to lyse red blood cells as per the manufacturer's instructions. Subsequently, the cells were digested with the digestive enzyme mixture again for 20 min at 37 °C while being shaken to isolate the cell mass into single cells. Cell suspensions were filtered through a 40 μm filter and centrifuged at 1000 rpm for 5 min. The cells were washed twice and resuspended in Phosphate Buffer Saline (PBS). After determining cell count and viability, the cells were diluted to a concentration of approximately 1000/μL.

### The scRNA-seq and data processing

The scRNA-seq experiment was conducted at CapitalBio Technology Co., Ltd. In brief, the single-cell suspension was loaded onto the 10X Genomics single-cell chip with the goal of capturing 3000 cells/chip. The library was prepared according to the recommended protocol of the 10X Genomics Single Cell 3ʹ Kit. The sequencing library was constructed using single-cell 3ʹ v2 chemistry (10 × Genomics) and sequenced on Illumina HiSeq X-10, generating 90 G data for each sample.

The raw sequencing data underwent processing by Cell Ranger (version 3.02), which is the official bundled software of 10X Genomics. We aligned the data to the human reference genome (GRCh38) using the default pipeline. Following this, we imported the Gene-Barcode matrics, which contain barcode information and expression counts, into the R package Seurat (version 4.2.0) [12]. In Seurat, we excluded certain cells based on their feature number, percentage of the mitochondrial transcript, and the counts of RNA. Specifically, we excluded cells that expressed fewer than 200 or more than 7500 genes, cells with mitochondrial transcript percentages greater than 25, and cells with RNA counts less than 200 or more than 20,000.

After data pre-processing, we employed the “SCTransform” method to normalize and identify the most variable features for each sample. “SCTransform” avoids some of the drawbacks associated with the standard normalization workflow, including the addition of pseudo-counts and logarithmic transformations. To more effectively mitigate batch effects across multiple samples, we employed the anchor-based integration approach implemented in Seurat 3. Specifically, we utilized the “FindIntegrationAnchors” and “IntegrateData” functions to integrate gene expression matrices from five distinct samples. To achieve linear conversion, the “ScaleData” function was applied to scale the data.

### Cell type identification

The top 3000 genes with high variability were extracted for principal component analysis (PCA), and the 30 most significant principal components were utilized for cluster analysis. The “FindClusters” function (resolution = 0.8) was employed to identify the clusters, which were then visualized using the Uniform Manifold Approximation and Projection (UMAP). The characterization of cell types was based on gene function and the expression of known markers in the CellMarker database [13]. Furthermore, the sub-clustering of neoplastic cells and macrophages was carried out by utilizing the aforementioned approach. The marker genes for each cluster were identified by the “FindClusters” function with a resolution of 0.1.

### Weighted gene co-expression network analysis (WGCNA)

In order to investigate the neoplastic cells, we conducted WGCNA using the ‘‘hdWGCNA’’ package in R. The analysis object was created using SetForWGCNA, with the gene selection parameter set to ‘‘variable’’. In the construction of ‘‘metaccells,’’ we employed a nearest neighbor parameter set to the default value of 25 and a soft threshold set to 12. By utilizing the "ConstructNetwork" functionality, a soft threshold of 12 was chosen, which represents the minimum value satisfying a scale-free topology fit of 0.8. When the specified criteria are met, this results in an elevation of connectivity strength among network edges, consequently leading to the formation of more densely interconnected gene modules. Taking into account the dataset's size, we employed the “ModuleExprScore” function and the “Ucell” method to compute scores for the top 50 genes in each module.

### Gene regulatory network analysis

We applied SCENIC with the pySCENIC package (0.12.1) to investigate the dominant transcription factors in distinct neoplastic clusters in Python (version 3.9.16). pySCENIC consists of three major processes. First, coexpression modules are inferred using a regression per-target approach (GRNBoost2). Next, the indirect targets are pruned from these modules using cis-regulatory motif discovery (cisTarget). The gene-motif ranking (10 kb around the transcription start site) was used as a guide to determine the search space around the transcription start site for transcription factor regulatory networks. Human gene-motif rankings are collected from https://resources.aertslab.org/cistarget/. Lastly, the activity of these regulons is quantified via an enrichment score for the regulon's target genes (AUCell). we aimed to establish specific associations between regulons and individual tumor cell clusters based on the regulon specificity score (RSS) criteria of RSS > 0.1 and RSS standard deviation > 3. Through this approach, we filtered and selected regulons showing significant specificity to each neoplastic cell cluster. Visualization was performed using the R packages “pheatmap” and “ggplot2”.

### Pseudotime trajectory analysis

All neoplastic and macrophage cells were chosen for analysis of the pseudotime trajectory. The R package ‘‘Monocle’’ was utilized for differentiation trajectory and pseudotime analysis. A ‘‘CellDataSet’’ was formed based on the Seurat objects for neoplastic and macrophage cells. The “newCellDataSet” function was applied to create an object with the parameter expressionFamily = negbinomial.size. Highly variable genes were chosen for analysis based on the “dispersionTable” using the monocle2 package. Genes with greater dispersion levels were selected using the "dispersionTable" function in Monocle. In the trajectory analysis, we used genes meeting the thresholds that mean_expression ≥ 0.1 and dispersion_empirical ≥ 1 * dispersion_fit identified by Monocle2 to sort cells in pseudo-time order. The reduceDimension() function using the parameters reduction_method = “DDRTree” and max_components = 2 was applied to reduce dimensions and the visualization functions ‘plot_cell_trajectory’ were used to plot the minimum spanning tree on cells. Genes that changed along with the pseudotime were calculated (*q*-val < 0.05) by the “differentialGeneTest” function and visualized with the plot_pseudotime_heatmap and the genes were clustered into subgroups according to the gene expression patterns. To identify the genes that separate cells into branches, the branch expression analysis modeling (BEAM) analysis were performed and genes resulting from the BEAM analysis with a *q*-value =  = 0 were separated into groups and visualized with the plot_genes_branched_heatmap function.

### Enrichment analyses

We conducted gene ontology (GO) and Kyoto Encyclopedia of Genes and Genomes (KEGG) enrichment analyses using the “clusterProfiler” package in R [13]. Both GO (adjusted P < 0.05) and KEGG (adjusted P < 0.05) enrichment analyses were performed on all genes. We then grouped the significantly enriched GO items in each dataset into the functional network within ClueGO v. 2.5.9 in Cytoscape v. 3.9.1, utilizing the default parameters of edge-weighted, force-directed, and BioLayout for CluePedia. We constructed a network of pathways by conducting GO enrichment analysis of genes using Cytoscape.

### Cell communication analysis

Intercellular communication was evaluated using CellChat 0.0.2, an R package, by assessing the expression of ligand-receptor pairs within cell clusters. For further analysis, the "Secreted Signaling" section in the database was selected, while cell types with fewer than 50 cells, such as oligodendrocytes, were filtered out. Additional interactions between different cell types were also investigated.

### Statistical analysis

Downstream data analysis of scRNA-seq was performed using Seurat, CellChat, Monocle, and pySCENIC packages, along with clusterProfiler for statistical analysis. Marker genes for each cluster were calculated using the two-sided Wilcoxon rank-sum test implemented in the Seurat R package to assess differential gene expression. Bonferroni correction was applied to adjust p-values based on the total number of features in the dataset. WGCNA involved calculating pairwise correlations of input features, determining topological overlap using soft-thresholded weighted correlations, and performing unsupervised clustering with the dynamic tree cut algorithm. Gene enrichment analysis utilized the Hypergeometric Test as the hypothesis test, with the Benjamini–Hochberg method (also known as FDR correction) applied for multiple testing correction of p-values. All statistical analyses, except for pySCENIC, were conducted using R (version 4.2.2). q-values or adjusted p-values less than 0.05 were considered statistically significant in this study.

### Multiplex immunofluorescence staining

Multiplex immunofluorescence staining was carried out by implementing the Opal Manual IHC Kit (Akoya Biosciences, MA, USA). The tissue samples of paraffin-embedded meningioma were sliced into thin sections with a thickness of 5 μm. These sections underwent a deparaffinization process in xylene, lasting for 15 min on two occasions, followed by a hydration process using ethanol with varying concentrations (100%, 95%, 85%, and 75%) with each lasting for 10 min. Subsequently, the sections were washed with TBST for 5 min.

After undergoing antigen retrieval via microwave heating in citrate buffer for a duration of 15 min, the sections were allowed to cool down at ambient temperature for 15–30 min. The sections were then washed twice with TBST for 5 min each. Next, the sections were exposed to primary antibody (anti-MIF: 1:200, abcam, ab55445; anti- CD74: 1:200, abcam, ab10839; anti- VIMENTIN: 1:400, CST, #5741) at room temperature for a period of 60 min and washed with TBST for 5 min thrice. Following this, the sections were incubated with the secondary antibody at room temperature for 15 min, and washed again with TBST for 5 min thrice. Opal 540/640/570 fluorescent dye staining was carried out for a duration of 10 min at room temperature, and the sections were washed with TBST for 5 min thrice.

The sections were subjected to antigen retrieval using citrate buffer and microwaved for 15 min. Bound antibodies were subsequently detached and the sections cooled at room temperature for 15–30 min. Following this, the sections were washed twice with TBST for 5 min each. Subsequently, the slices were incubated in DAPI staining solution at room temperature for 5 min to stain the nuclei. After incubation, the sections were washed with TBST for 5 min followed by washing with ultrapure water for 5 min. The slices were then mounted with anti-fade mounting medium and stored in the dark. Finally, the stained slices were imaged by scanning with the Akoya PhenoImager.

### Data availability

Five single-cell transcriptome datasets of meningioma have been deposited in the Genome Sequence Archive of Beijing Institute of Genomics, Chinese Academy of Sciences, with the accession number PRJCA017724 [14], These datasets are publicly available for access at https://bigd.big.ac.cn/gsa-human/browse/HRA004857.

Additionally, there is a publicly available dataset, GSE183655, which was published in 2022 and generated using the 10X Genomics platform for single-cell data (https://www.ncbi.nlm.nih.gov/geo/query/acc.cgi?acc=GSE183655). This dataset comprises eight meningioma samples obtained from six patients, two of which had two samples analyzed respectively. One sample was collected from the tumor bulk, while the other was obtained from the brain-tumor interface (BTI).

## Results

### Cell composition in distinct subtypes of meningioma.

**Fig. 1:**
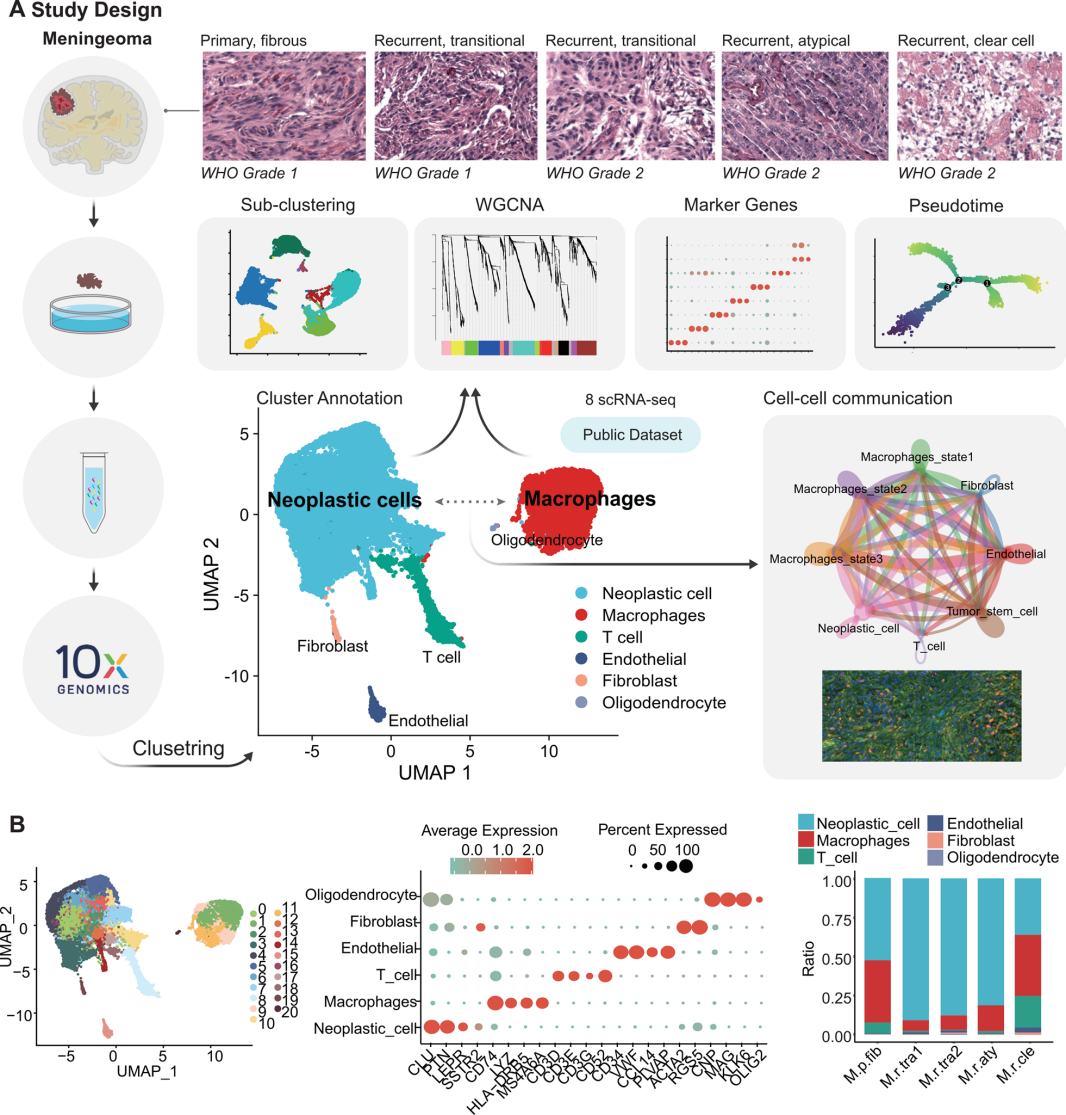
Study design and meningioma landscape. **A** The Meningioma specimens were collected during surgical resection of brain tumors and histologically classified into five distinct pathological types or WHO grades using HE staining. The samples were subjected to scRNA-seq analysis, and after data integration, clustering, and cell type identification, we identified six distinct cell types. We further explored the biological significance and communication relationships of important cell populations during meningioma tumorigenesis using cell type-specific clustering, WGCNA, pseudotime analysis, and cell communication analysis. Additionally, we performed the same analyses on publicly available datasets. **B** Uniform manifold approximation and projection (UMAP) plot of 23,695 cells (left), color-coded by associated cluster. Each point depicts a single cell. All cells were identified as six different cell types, and corresponding marker genes were determined for each cell type (middle): neoplastic cells (*CLU*, *PTN*, *LEPR*, and *SSTR2*); macrophages (*HLA-DRB5*, *CD74*, *MS4A6A*, and *LYZ*); T cells (*CD3D*, *CD3E*, *CD3G*, and *CD52*); endothelial cells (*CD34*, *VWF*, *CCL14*, and *PLVAP*); fibroblasts (*ACTA2* and *RGS5*); and oligodendrocytes (*CNP*, *MAG*, *KLK6*, and *OLIG2*). Scaled color bar = average expression, Size of the point = percent expressed. Proportions of the six cell types in the sample are shown on the right

As shown in Fig. 1A, our research encompassed five meningioma specimens for subsequent scRNA-seq analysis, with clinical details elucidated in Table 1 (see Methods). According to the primary/recurrent meningioma and pathological type (the same pathological type was further graded according to WHO), the five samples were respectively named as “M.p.fib”, “M.r.tra1”, “M.r.tra2”, “M.r.aty”, “M.r.cle”. The HE staining results of these five meningioma samples were congruent with their respective pathological diagnoses (Fig. 1A). HE staining typically revealed variations in cellular morphology and size, with neoplastic cells exhibiting features such as nuclear division and heterogeneity that differ significantly from those of normal cells. In total, 23,695 cells were obtained after data filtering and quality control. We resolved 21 cell clusters using cell clustering analysis (Methods, Fig. 1B-left). Among these clusters, six cell types were identified based on known marker genes: neoplastic cells (16,901), including *CLU*, *PTN*, *LEPR*, and *SSTR2*; macrophages (4795), including *HLA-DRB5*, *CD74*, *MS4A6A*, and *LYZ*; T cells (1430), including *CD3D*, *CD3E*, *CD3G*, and *CD52*; endothelial cells (351), including *CD34*, *VWF*, *CCL14*, and *PLVAP*; fibroblasts (181), including *ACTA2* and *RGS5*; and oligodendrocytes (37), including *CNP*, *MAG*, *KLK6*, and *OLIG2* (Fig. 1B-middle, Additional file 1: Figure S1A–C, Additional file 11: Table S1). In our dataset, we observed high expression of the well-established meningioma marker genes *LEPR* and *SSTR2* in meningioma neoplastic cells[15–18]. Furthermore, the genes *CLU* and *PTN*, which are known to be highly expressed in other neoplastic cells, were also found to be commonly expressed in meningioma neoplastic cells [19, 20]. Here, neoplastic cells had the highest proportion (71.3%) of cells in samples, followed by macrophages (20.2%). T cells accounted for only 6.2%, and their counts showed great individual variability. More than 77.3% of all T cells originated from M.r.cle, subtype “clear cell” (Fig. 1B-right, Additional file 11: Table S1).

We conducted the analysis with the same procedure on a total of eight tissue samples from six patients in the public GSE183655 dataset, albeit lacking clinical information for subtype comparison. It had 45,238 cells being partitioned into 23 distinct cell clusters, which were further categorized into six distinct cell types with their marker genes identified, as illustrated in Additional file 2: Figure S2A–C, 2E–F. Consistent with our results, the public dataset exhibited a higher prevalence of neoplastic cells, followed by macrophages, while T-cell counts varied significantly among samples (Additional file 2: Figure S2D, Additional file 11: Table S1).

### Neoplastic cell heterogeneity: neoplastic cell re-clustering and WGCNA

**Fig. 2:**
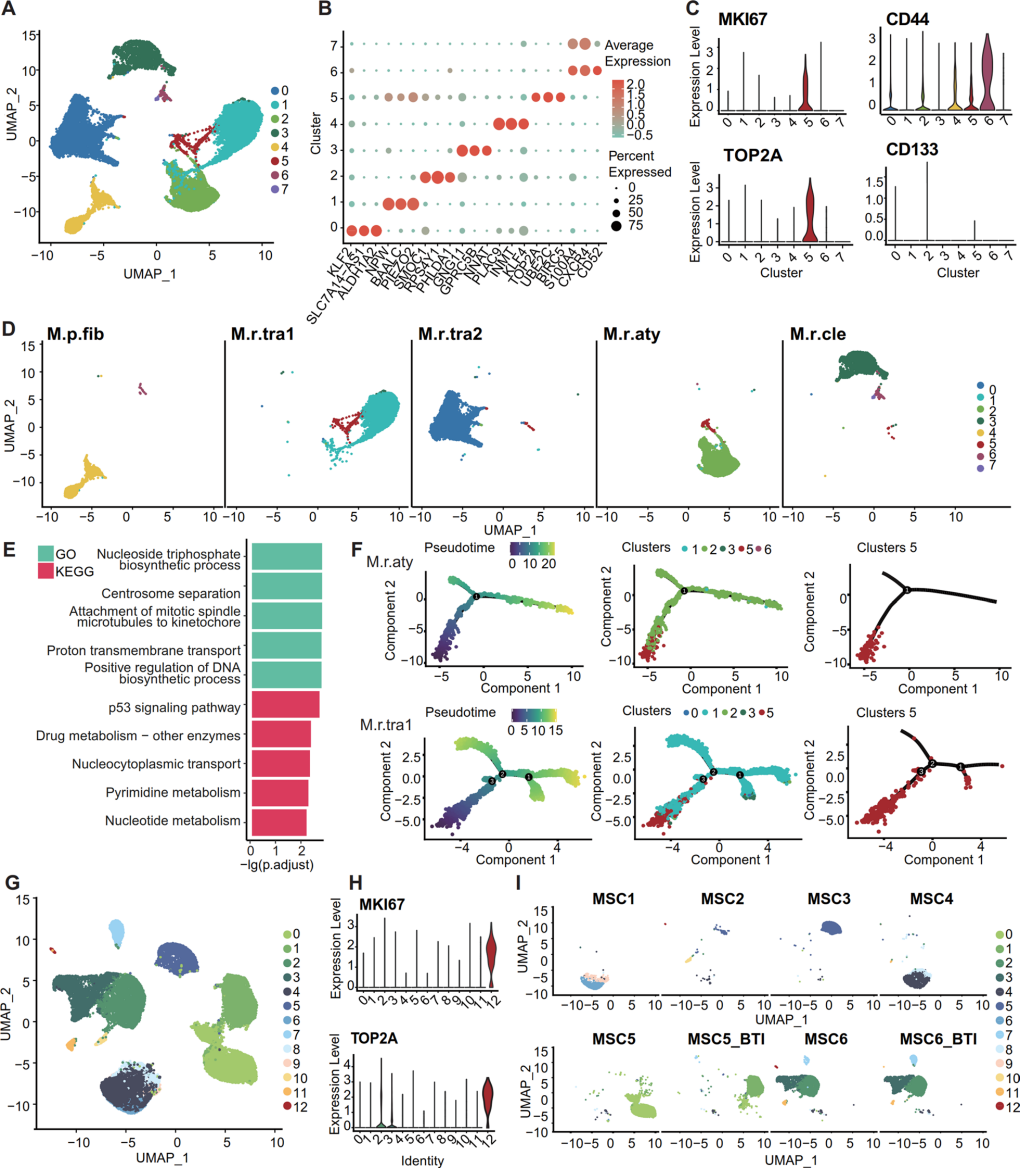
Neoplastic cells subclustering and pseudotime analysis. **A** UMAP plot of 16,901 neoplastic cells, color-coded by associated cluster. Neoplastic cells were re-clustered into eight subclusters (N0-N7). Each point depicts a single cell. **B** Marker genes for neoplastic cells in subclusters N0-N7. Scaled color bar = average expression, size of the point = percent expressed. **C** Expression of *MKI67*, *TOP2A*, *CD44*, and *CD133* in neoplastic cells. **D** Separation of neoplastic cell subclusters in each sample. **E** GO and KEGG enrichment analysis of marker genes in N5 neoplastic cells (Top 5, p adjusted < 0.05). **F** Pseudotime analysis of M.r.aty and M.r.tra1 neoplastic cells. The pseudotime evolution time relationship is shown. Scaled color bar = pseudotime (left). Distribution of neoplastic cell on the pseudotime map (right). Each cell in the branched pseudotime trajectory was colored by its pseudotime value and its Seurat clusters. **G** In a public dataset, neoplastic cells were re-clustered into 13 subclusters. **H** Expression of *MKI67* and *TOP2A* in neoplastic cells from the public dataset. **I** Separation of tumor cell subclusters in each sample from the public dataset. Tumor cell distribution showed similarity in tissues from the same sample (e.g., MSC6 and MSC6_BTI). Of these samples, MSC5 (tumor bulk) and MSC5_BTI (brain-tumor interface) were obtained from one patient, while MSC6 (tumor bulk) and MSC6_BTI (brain-tumor interface) from another patient. Each of the remaining samples was from distinct patients

**Fig. 3:**
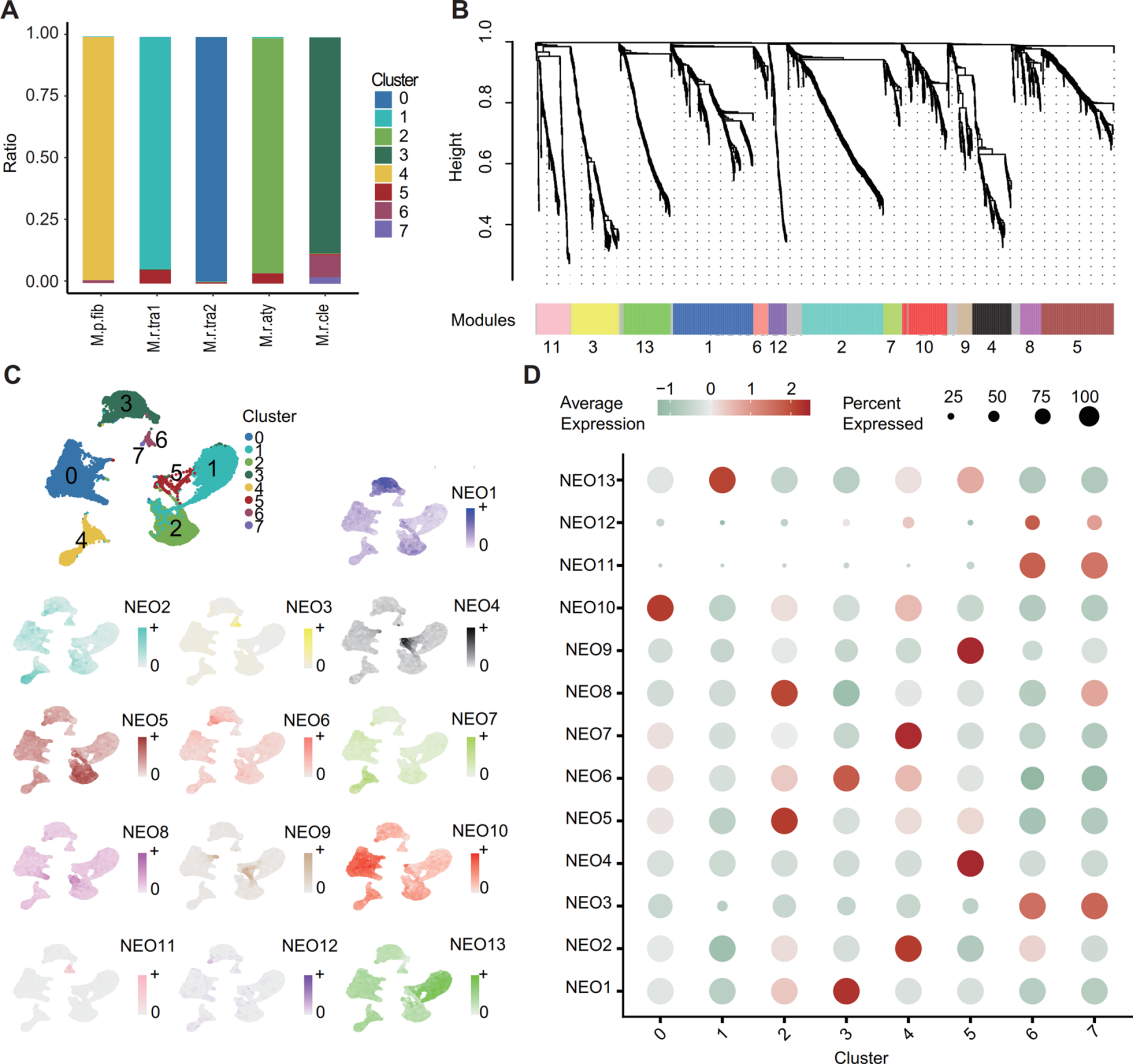
WGCNA analysis of neoplastic cells. **A** Proportions of different subclusters of neoplastic cells in the samples. **B** Gene co-expression modules identified by WGCNA analysis of neoplastic cells. Thirteen modules (NEO1-NEO13) were identified, with each module represented by a different color except for the gray module. **C** Expression profiles of the top 50 genes in each of the 13 modules in the neoplastic cell UMAP. **D** Expression of the genes in the 13 modules across different subpopulations of neoplastic cells. Scaled color bar = average expression, size of the point = percent expressed

We performed further subclustering of our neoplastic cells (n = 16,901, Methods), resolving 8 cell clusters (N0-N7, Fig. 2A). Each cluster was featured by distinct marker genes (Fig. 2B). After batch effect correction (Methods), neoplastic cells from different samples still formed distinct clusters (Fig. 2D, Fig. 3A), suggesting significant heterogeneity among meningioma neoplastic cells in our samples.

Nevetheless, N5-neoplastic cells were present in various subtypes, among which M.r.aty and M.r.tra1(atypical and transitional) have the highest proportion. Particularly, They show high expression of cell proliferation-related genes *MKI67* and *TOP2A*, but tumor stem cell marker genes *CD44* and *CD133* were not highly expressed (Fig. 2C). GO enrichment analysis of all the marker genes in N5-neoplastic cells demonstrated that (Methods), the most significant biological processes were related to cell proliferation and division, such as ‘‘triphosphate nucleotide biosynthetic process’’ (adjusted P = 0.001758, Hypergeometric Test), ‘‘centrosome separation’’ (adjusted P = 0.001758, Hypergeometric Test), and ‘‘attachment of spindle microtubules to kinetochores’’ (adjusted P = 0.001758, Hypergeometric Test, Fig. 2E). Meanwhile, their KEGG enrichment analysis found the most significant pathway was the “p53 signaling pathway” (adjusted P = 0.002221, Hypergeometric Test, Fig. 2E). Therefore, N5-neoplastic cells could represent a small fraction of highly proliferative cells within the tumor. Pseudotime analysis of neoplastic cells in M.r.aty and M.r.tra1 revealed that N5-neoplastic cells are primarily located at the beginning of the pseudotime differentiation branch and in an early differentiation stage (Fig. 2F).

Similarly, neoplastic cells from the public database were also clustered into 13 clusters (S1-S13, as shown in Fig. 2G). Neoplastic cells from different sites of the same patient cluster together, while those from different patients form distinct clusters, providing evidence for the significant inter-individual heterogeneity observed in our meningioma tumor cells (Fig. 2K). Similar to the N5 -neoplastic cells we discovered, this public dataset also contains a cluster of cells with high expression of *MKI67* and *TOP2A* (MSC6-S12, Fig. 2I and Additional file 3: Figure S3B). The difference lies in the tumor stem cell marker genes, where *CD133* was not highly expressed, but *CD44* showed high transcription. This finding warrants further exploration of the inter-sample differences in the expression of these two markers with additional samples. Enrichment analysis revealed that highly expressed genes in MSC6-S12 were significantly associated with "chromosome segregation" (adjusted P = 1.87E-30, Hypergeometric Test) and ‘‘cell cycle’’ (adjusted P = 3.88E-15, Hypergeometric Test, Additional file 3: Figure S3C). Furthermore, pseudotime analysis indicated that MSC6-S12 was located at the branch end of the pseudotime trajectory and in the early stage of differentiation (Additional file 3: Figure S3D).

To show the biological features of the neoplastic cell cluster, we performed WGCNA to identify their 13 gene co-expression modules (Fig. 3B, Additional file 12: Table S2). The gene scores of each module demonstrated differential expression among the neoplastic cell subclusters (adjusted P < 0.05, two-sided Wilcoxon rank-sum test, Fig. 3C–D). Furthermore, the enrichment outcomes pertaining to genes in each module exhibited a broad spectrum of diversity, encompassing immune-related signaling pathways, Wnt signaling pathways, metabolic-related pathways, and response to decreased oxygen levels, among others (Table 2, Additional file 13: Table S3). The variety of enrichment outcomes between modules exhibits the heterogeneity of neoplastic cells (Table 2). The public dataset also revealed extensive biological processes enriched in module genes, including central nervous system development and differentiation, immune-related signaling pathways, ion and substance transport and regulation, response to low oxygen levels, and metabolism-related pathways (Additional file 3: Figure S3E–G, Additional file 15: Table S5). These findings exhibited strong concordance with our own data results. In addition, we further identified 99 activated regulons in meningioma neoplastic cells demonstrating sample specificity (Additional file 4: Figure S4B).

**Table 2:** Seurat clusters and WGCNA module-sample list for neoplastic cells, with GO enrichment summary

WGCNA NEO	Seurat Cluster	Sample ID	Enrichment analysis features
NEO 1	Cluster 3	M.r.cle	Regulation of metabolism and neuronal development
NEO 2	Cluster 4	M.p.fib	Cell development and glucocorticoid response
NEO 3	Cluster 6 and 7	M.r.cle	Immune-related signaling pathways
NEO 4	Cluster 5	M.r.aty and M.r.tra1	Mitosis and DNA replication
NEO 5	Cluster 2	M.r.aty	Immune-related signaling pathways
NEO 6	Cluster 3	M.r.cle	Regulation of Wnt signaling pathway
NEO 7	Cluster 4	M.p.fib	Cell development and metabolic regulation
NEO 8	Cluster 2	M.r.aty	Regulation of cell adhesion and response to reduced blood oxygen levels
NEO 9	Cluster 5	M.r.aty and M.r.tra1	Mitosis and DNA replication
NEO 10	Cluster 0	M.r.tra2	Extracellular structures and epithelial cell migration
NEO 11	Cluster 6 and 7	M.r.cle	Immune-related signaling pathways
NEO 12	Cluster 6 and 7	M.r.cle	Immune-related signaling pathways
NEO 13	Cluster 1	M.r.tra1	Metabolic related pathways

Notably, the N5 subgroup exhibited higher gene scores in the NEO4 and NEO9 modules (Methods), which were significantly enriched in biological processes related to cell cycle and DNA replication (Table 2, Additional file 13: Table S3). On the other hand, the GSE-NEO9 module, which is associated with cell division processes such as ‘‘mitotic nuclear division’’ (adjusted P = 1.55E-50, Hypergeometric Test, Additional file 14: Table S4, Additional file 15: Table S5), exhibited higher gene scores in the MSC6-S12 subgroup. Within each neoplastic cell cluster, we observed elevated regulon specificity scores for E2F1 and E2F7 in N5-neoplastic cells, indicating enhanced transcriptional activity (Additional file 4: Figure S4A). The E2F family of transcription factors plays a crucial role in cell cycle regulation and DNA replication [21, 22].

### Two divergent cell fates of macrophages

**Fig. 4:**
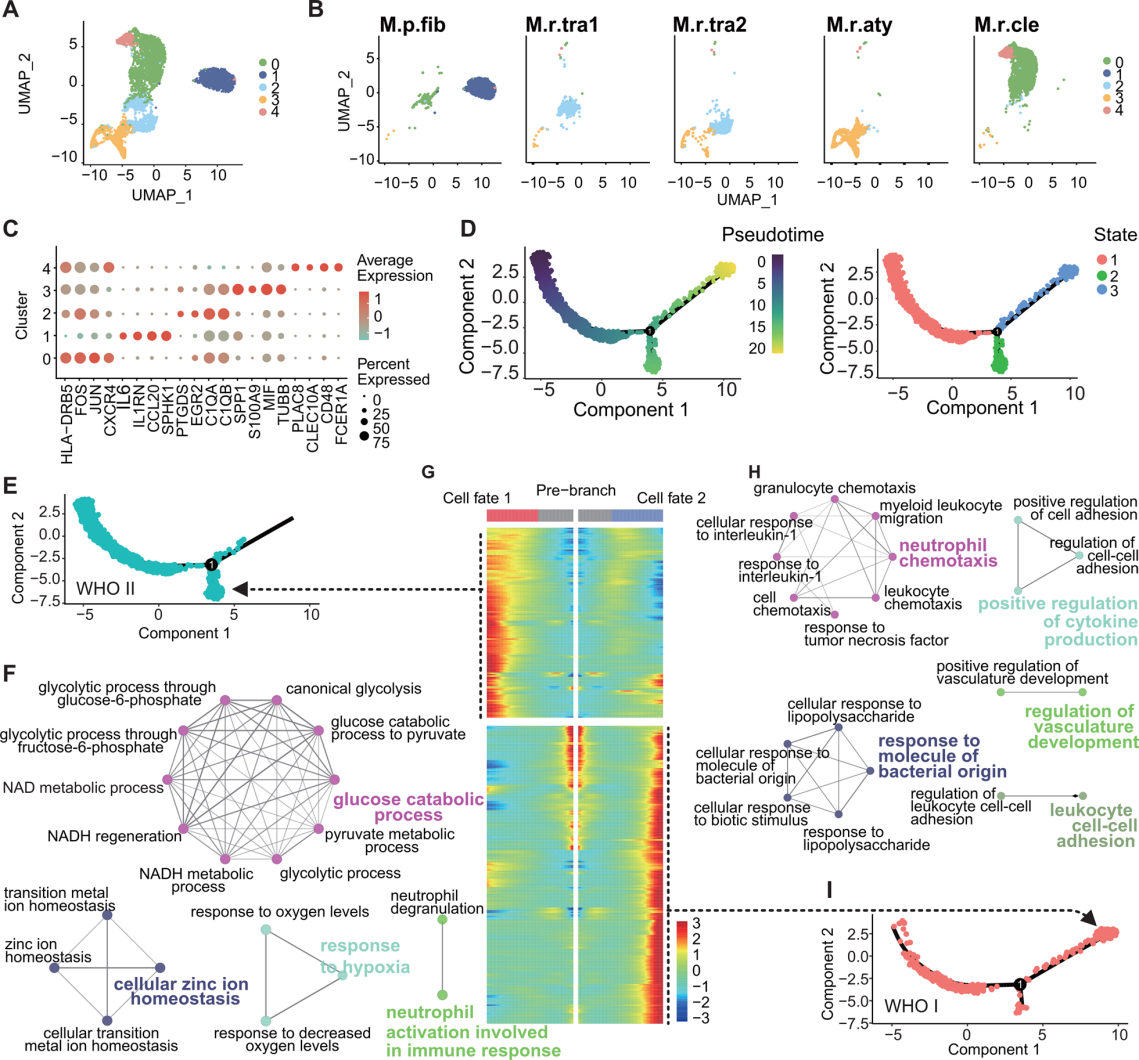
Subclustering and pseudotime analysis of macrophages in meningiomas. **A** UMAP plot of 4795 macrophages, color-coded according to their associated clusters. Macrophages were re-clustered into five subclusters. Each point represents a single cell. **B** Macrophage subclusters were separated across different pathological types of meningiomas and showed similar distribution in samples with the same pathological type (M.r.tra1 and M.r.tra2). **C** Marker genes for each macrophage subcluster. Scaled color bar = average expression, size of the point = percent expressed. **D** Pseudotime analysis of macrophages. Scaled color bar represents the pseudotime (left). Distribution of macrophage pseudotime states (right). Each cell in the branched pseudotime trajectory was colored by its pseudotime value and its Seurat clusters. **E** Distribution of WHO grade II meningioma macrophages in pseudotime analysis, mainly in state1 and state3. **F** Grouped network of enriched GO terms for high-expression genes in cell fate 1. **G** Heatmap of high-expression genes for different cell fates in pseudotime analysis. Scaled color bar indicates the average expression. The high-expression gene set for cell fate 1 is predominantly located on the left side of the heatmap, while that for cell fate 2 is concentrated on the right side. **H** Distribution of WHO grade I meningioma macrophages in pseudotime analysis, mainly in state1 and state2. **I** Grouped network of enriched GO terms for high-expression genes in cell fate 2

Within the tumor microenvironment (TME) of meningiomas, macrophages are ubiquitous and account for the majority of immune cells (n = 4759). These cells were subsequently classified into five distinct clusters based on their respective marker genes (Fig. 4A, C). Notably, clear heterogeneity among macrophages from different pathological types was observed, with macrophages from transitional meningiomas (M.r.tra1 and M.r.tra2) predominantly grouping into Cluster 2, while macrophages from other histological types clustered differently (Fig. 4B).

We conducted pseudotime trajectory analysis on macrophages in the TME of meningiomas (n = 4759) and categorized them into one node and three states (Fig. 4D). State 1 constituted the largest proportion (66.9%), followed by State 3 (20.2%) and State 2 (12.9%) (Additional file 16: Table S6). A heatmap of differential pseudotime analysis depicted genes that were highly expressed in the two distinct cell fates: State 2 corresponding to Cell fate 1 and State 3 corresponding to Cell fate 2 (Fig. 3G, Additional file 17: Table S7). Notably, macrophages in WHO grade 1 meningioma were more likely to cluster in State 3/Cell fate 2, while macrophages in WHO grade 2 meningiomas were more likely to cluster in State 2/Cell fate 1 (Fig. 4E, I). The two distinct functional roles of macrophages in meningiomas were further emphasized by GO enrichment analysis, where highly expressed genes in State 2/Cell fate 1 were associated with "glucose catabolic process," (adjusted P = 3.13E-05, Hypergeometric Test), "cellular zinc ion homeostasis" (adjusted P = 3.45E-05, Hypergeometric Test), and "response to hypoxia" (adjusted P = 3.86E-07, Hypergeometric Test) (Fig. 4F). In contrast, highly expressed genes in State 3/Cell fate 2 were associated with ‘‘neutrophil chemotaxis’’ (adjusted P = 5.01E−12, Hypergeometric Test), ‘‘response to molecule of bacterial origin’’ (adjusted P = 4.41E−15, Hypergeometric Test), and ‘‘positive regulation of cytokine production’’ (adjusted P = 2.68E−12, Hypergeometric Test) (Fig. 4H). Although the classical M1 and M2 macrophage marker genes failed to clearly classify subclusters of macrophages into two categories (Additional file 5: Figure S5A–B), macrophages in State 2/Cell fate 1 were more abundant in WHO grade II meningiomas and likely promote tumor development, similar to M2-type anti-inflammatory macrophages. Macrophages in State 3/Cell fate 2, on the other hand, preferentially occurred in WHO grade I meningiomas and played a pro-inflammatory role that benefited cancer cell clearance, similar to M1-type pro-inflammatory macrophages.

In the publicly available dataset, a cohort of macrophages (n = 6448) was further partitioned into 10 distinctive clusters, each evincing unique marker gene expression (Additional file 6: Figure S6A, C). Consistent with our own data, macrophages exhibited notable heterogeneity and could not be definitively classified into M1 or M2 subtypes solely based on their marker gene profiles (Additional file 6: Figure S6B, Additional file 7: Figure S7A, B). We ascertained that the biological process of "response to decreased oxygen levels’’ (adjusted P = 1.13E-3, Hypergeometric Test) was enriched solely in early-stage genes within the pseudotime trajectory analysis, while immune-related processes, such as ‘‘leukocyte cell–cell adhesion’’ (adjusted P = 4.92E-13, Hypergeometric Test), were predominantly present in the late-stage genes (Additional file 6: Fig. S6F–J).

### Cell communication: macrophage migration inhibitory factor (*MIF*) and *CD74* interaction in meningiomas

**Fig. 5:**
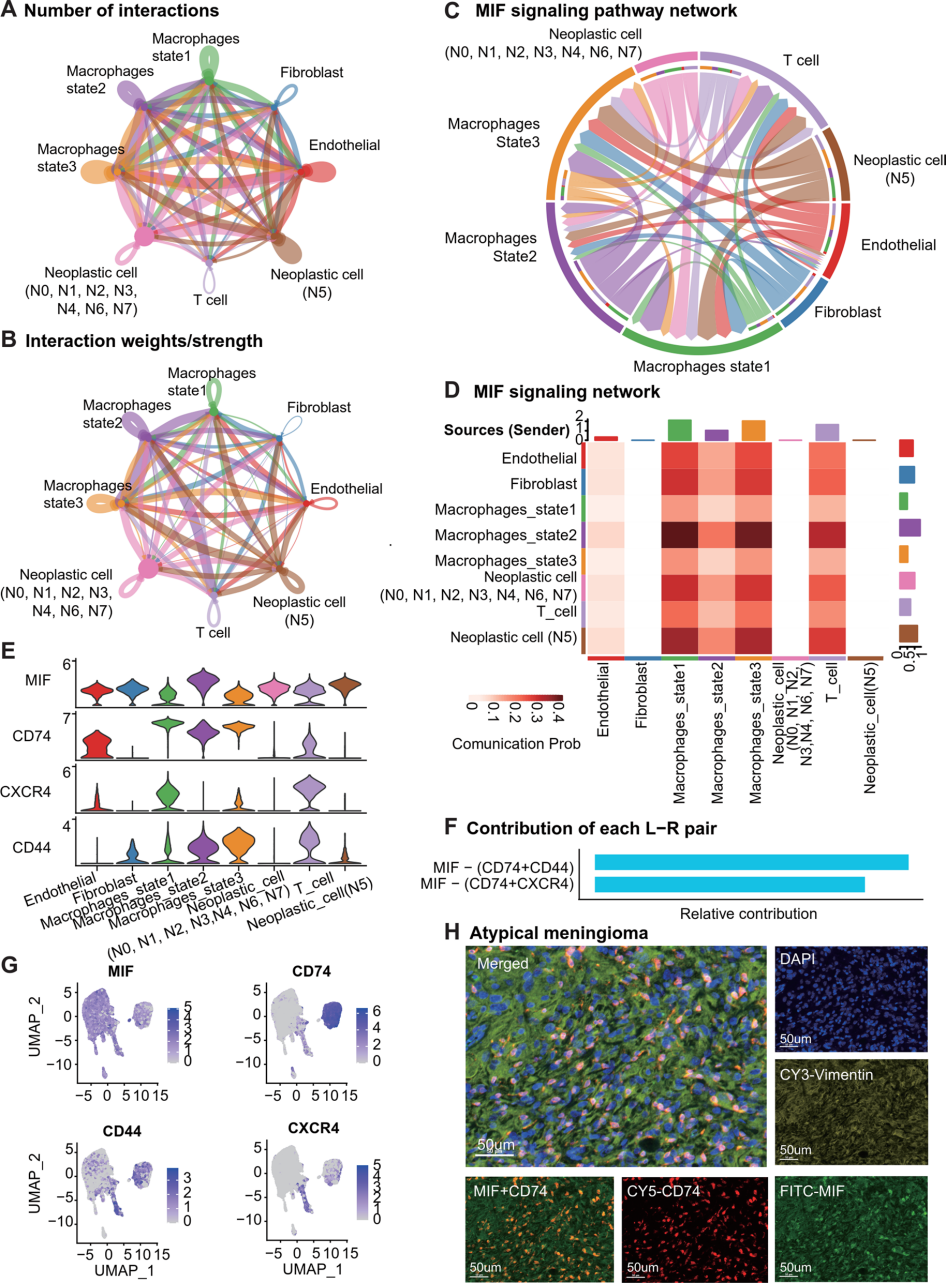
Intercellular communication analysis in meningiomas. **A** Number of interactions network among cells. The thickness of the lines represents the number of interactions. **B** Interaction weight network among cells. The thickness of the lines represents the interaction weight. **C** Chord plot showing the inferred intercellular communication network of *MIF* signaling. **D** Heatmap of communication probability in *MIF* signaling, scaled color bar = Communication Probability. **E** Violin plots of ligand and receptor genes (*MIF*, *CD74*, *CXCR4*, and *CD44*) expression in cells in the MIF signaling. **F** Ligand-receptor pairs included in the *MIF* signaling and their relative contribution. **G** UMAP plot showing cells expressing *MIF*, *CD74*, *CXCR4*, and *CD44* colored. **H** Multiplex immunofluorescence staining of atypical meningioma, *MIF* (red fluorescence), *CD74* (green fluorescence), *Vimentin* (yellow fluorescence), and DAPI (blue fluorescence). Scale bar, 50 μm

We extracted N5-neoplastic cells from the entire neoplastic cell population and introduced the three distinct states of macrophages to further elucidate the intricate communication relationships between cells. We quantified and depicted the total number and strength of intercellular communication (Fig. 5A, B). In our dataset, we identified a total of 33 significant signaling, including *MIF*, *SPP1*, *MK*, *PTN*, *VISFATIN*, *GALECTIN*, *CCL*, *COMPLEMENT*, *ANNEXIN*, *VEGF*, *CXCL*, *TNF*, *GAS*, *ANGPT*, *GRN*, *PDGF*, *PARs*, *TWEAK*, *PROS*, *TGFb*, *EDN*, *CSF*, *EGF*, *CALCR*, *OSM*, *SEMA3*, *BMP*, *FGF*, *CX3C*, *ANGPTL*, *IL6*, *WNT*, and *APELIN*. We further summarized the interactions between ligands and receptors among all cell types (P < 0.01, Permutation Test, Additional file 8: Figure S8A). The functions of these signals are diverse and complex, mostly associated with tumor growth, metastasis, recurrence, anti-apoptosis, tumor angiogenesis, and immunity, and the specific functions of each signal can be found in Additional file 18: Table S8. The most potent signaling identified in our analysis was the MIF signaling (P < 0.01, Permutation Test, Fig. 5C, D), with the receptor-ligand pairs MIF-(CD74 + CXCR4) and MIF-(CD74 + CD44) (Fig. 5F).

*MIF* expression was nearly ubiquitous across all cell types, while *CD74*, *CD44*, and *CXCR4* were expressed in macrophages, T cells, and endothelial cells (Fig. 5E, G). Consistent with our cell communication analysis, all cells in the MIF signaling emitted secretion signals, but only macrophages, T cells, and endothelial cells received secretion signals. N5-neoplastic cells exhibited a more profound impact on macrophages compared to other neoplastic cells. Our findings are in agreement with the detection of MIF-CD74 interaction in cell communication within public datasets. The majority of receptor cells remained macrophages, and high-growth neoplastic cells had a more significant influence on macrophages compared to other neoplastic cells (Additional file 9: Figure S9). In the three macrophage states, we observed that macrophages in state 1/pre-branch and state 3/cell fate 2 were in a similar state, and they received relatively higher signal intensity than state 2/cell fate 1.

**Fig. 6:**
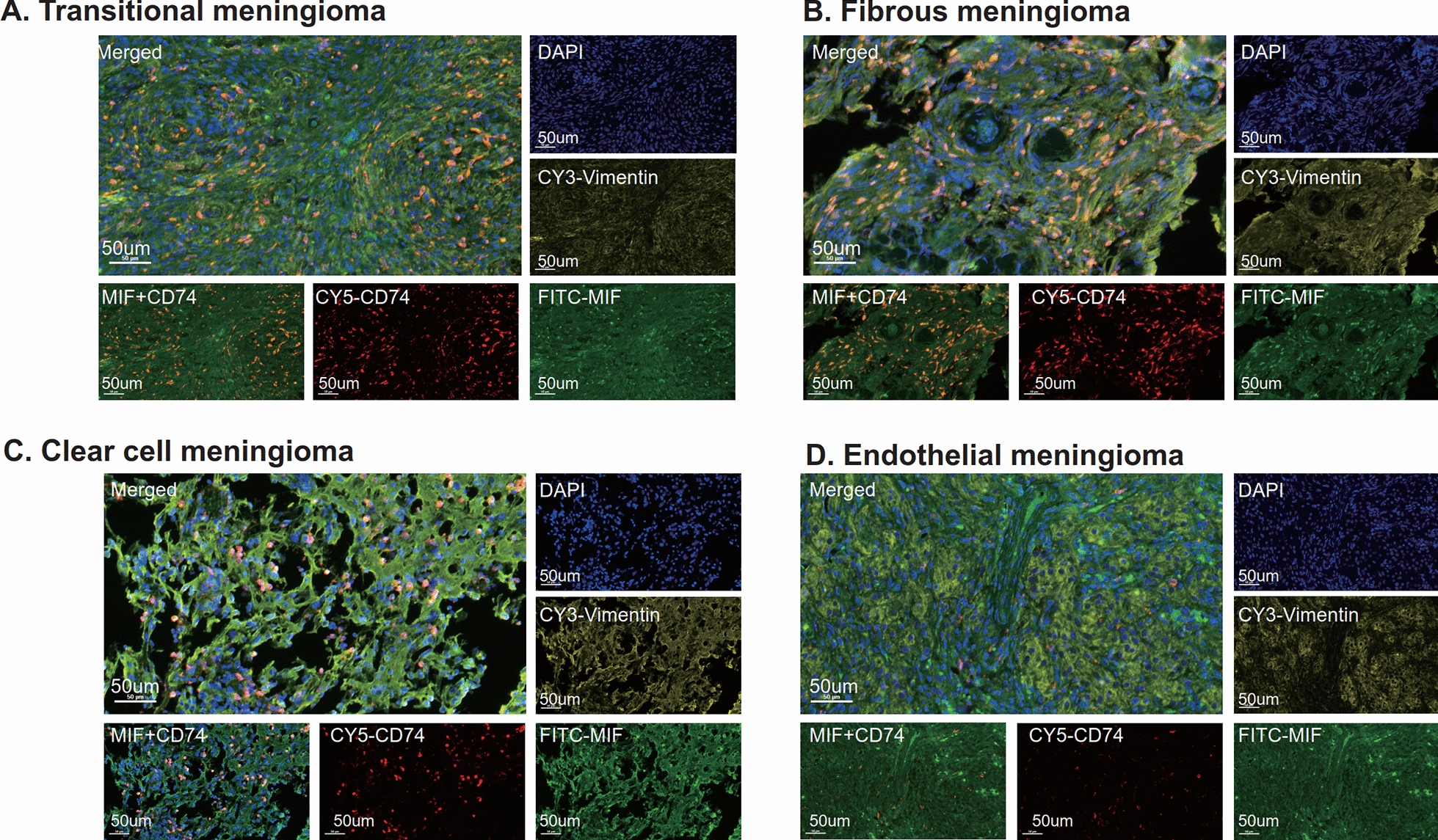
Multiple immunofluorescence staining of different pathological types of meningioma tissues. **A** Multiple immunofluorescence staining of transitional meningioma. **B** Multiple immunofluorescence staining of fibrous meningioma. **C** Multiple immunofluorescence staining of clear cell meningioma. **D** Multiple immunofluorescence staining of endothelial meningioma. The staining includes *MIF* (green fluorescence), *CD74* (red fluorescence), *Vimentin* (yellow fluorescence) and DAPI (blue fluorescence). Scale bar, 50 μm

In order to investigate the extensive MIF-CD74 interactions in meningiomas, immunofluorescence staining was performed on five distinct types of meningiomas, including DAPI, MIF, CD74, and the meningioma marker Vimentin. Each meningioma subtype exhibited distinctive pathological characteristics. Immunofluorescence staining results revealed the presence of MIF (green fluorescence) and CD74 (red fluorescence) in atypical, transitional, fibrous, clear cell, and endothelial subtypes of meningiomas (Figs. 5H, 6). MIF demonstrated broad expression both in the cytoplasm and nucleus, whereas CD74 was predominantly expressed in the nucleus of immune cells. In order to ensure the robustness of the broad expression of MIF and CD74 observed within meningiomas, a subsequent investigation was conducted involving an additional cohort of ten meningioma cases. This extended cohort comprised four non-typical meningiomas, two transitional meningiomas, two variant meningiomas, and two endothelial meningiomas (Additional file 10: Figure S10). The evidence for the widespread expression of MIF and CD74 within meningiomas was further fortified through immunofluorescence staining of the aforementioned ten meningioma samples with MIF (green fluorescence) and CD74 (red fluorescence).

## Discussion

We conducted single-cell transcriptomic analysis on five distinct meningioma specimens with varying pathological subtypes or WHO grades. The neoplastic cells constitute the preponderant cellular population in meningiomas. Analogous to other types of tumors, meningiomas exhibit pronounced heterogeneity [23, 24]. Our dataset demonstrated distinct neoplastic cell populations from different samples that occupied separate clusters, which were well-separated in UMAP analysis. Tumor stem cells (TSCs) constitute only a minute fraction, ranging from approximately 0.05% to 3%, of the heterogeneous tumor masses [25]. As one of the determinants of tumor heterogeneity [26, 27], TSCs are of great significance. Notably, the N5 cluster of neoplastic cells in our dataset exhibited a lower cell count (< 3%) but displayed elevated expression levels of the specific markers *MKI67* and *TOP2A*. Evaluation of *MKI67* expression has been suggested to facilitate prognostic prediction and to aid in assessing the potential for increased growth in meningiomas [28]. Our results of the enrichment analysis of N5-neoplastic cell marker genes and pseudotime analysis of the cluster support its highly proliferative features. In N5-neoplastic cells, we also observed specificity in the E2F family of transcription factors, which are involved in cell cycle regulation and associated with enhanced DNA replication and cell proliferation activity [21, 22, 29, 30]. However, traditional TSC markers such as *CD44* and *CD133* are insignificantly expressed in N5-neoplastic cells. Intriguingly, the negligible expression of *CD133* in our dataset and publicly available datasets is in contrast to some previous findings in meningiomas research [31–33].

Within TME, macrophages exceed the number of tumor-infiltrating lymphocytes (T cells), as is consistent with our publicly available data and some other meningioma studies [8, 34]. The composition of infiltrating immune cell populations, however, varies not only across different tumor types but also within the same tumor or at different time points (e.g., at diagnosis versus relapse) [35]. Our samples indicated that macrophages of the same pathological type are clustered together, suggesting that macrophages may exhibit similar features in the same type of meningioma. While the expression of marker genes associated with M1 and M2 macrophages did not clearly categorize the macrophages in our data into two distinct subtypes, pseudotime trajectory analysis revealed two opposing macrophage fate states in meningiomas, which are distributed differently in WHO grade I and II meningiomas. These two macrophage fate states have opposing effects on tumors, with one promoting and the other inhibiting, similar to the functions of M2 and M1 macrophages. The emergence of a biological process indicating “response to decreased oxygen levels” in the context of pseudotime analysis of public datasets of macrophages suggests that macrophages are undergoing a potential process that promotes tumor development.

Except for meningiomas, MIF exhibits significantly elevated expression across various cancer types, indicating its potential as a diagnostic biomarker for tumor invasion and recurrence [36, 37]. Previous studies have demonstrated that MIF regulates multiple signaling pathways, such as nuclear factor-kappa B (NF-κB), ERK1/2, and activator protein-1 (AP-1), or modulates cellular responses through binding to the CD74/CD44 receptor complex. Meanwhile, CD74 plays a role in tumor occurrence. In skin cancer models of WT, P1G-MIF, and MIF knockout mice revealed that the P1G-MIF group (lacking tautomerase activity and binding to CD74) displayed intermediate tumor incidence between the WT and knockout groups [38]. Knockdown of CD74 in gastric cancer cells significantly reduced cell proliferation [39]. Similar results were observed in hepatocellular carcinoma, where CD74 deficiency led to reduced proliferation and a decreased tumor number in CD74 − / − mice compared to the wild-type controls [40]. Directly blocking CD74 represents one approach to inhibit all MIF-CD74 signal transduction.

Here, our intercellular communication analysis revealed the possible critical role of the MIF-CD74 interaction in meningiomas. In other tumors, MIF-CD74 exerts effects that promote tumor growth, inhibit apoptosis, induce tumor angiogenesis, and facilitate immune evasion [36, 40–43]. Therefore, the MIF-CD74 signaling pathway may impact tumor cell proliferation and survival in meningioma. Particularly, our data indicated that MIF may mediate the activation of CD74-positive cells, primarily macrophages. State 1/pre-branch and state 3/cell fate 2 macrophages appear to be similar, exhibiting stronger signal intensity compared to state 2/cell fate 1. These results suggest that *MIF*-*CD74* may potentially modulate macrophage state, leading them towards a state that favors the promotion of tumor development [44]. Additionally, compared to other tumor cells, N5-neoplastic cells with high proliferative characteristics have stronger effects on state 1/pre-branch and state 3/cell fate 2 macrophages. Simultaneously, research has also revealed that TSCs demonstrate a more intricate and potent modulation of macrophages compared to their ordinary tumor cell counterparts. TSC-derived factors have the ability to stimulate macrophage activation and polarization towards a pro-tumor phenotype, fundamentally altering the functional state of macrophages [45–49]. These are also consistent with a stronger effect of N5-neoplastic cells on macrophages. We have identified a common mechanism in different pathological subtypes (including atypical, transitional, fibrous, and clear cell) of meningiomas, namely the ubiquitous presence of *MIF* and *CD74*-positive macrophage interactions. This result has been confirmed through both public datasets and immunofluorescence staining.

Currently, experimental evidence has demonstrated the beneficial effects of blocking MIF-CD74 in tumor therapy, suggesting the potential importance of targeting this signaling pathway in meningioma treatment. In the future, further investigations should explore the specific molecular mechanisms of the MIF-CD74 signaling in meningioma tumor growth, proliferation, and the formation of tumor-promoting tumor microenvironments. Considering the potential clinical significance of our research results, we propose conducting large-scale animal experiments to verify the feasibility and efficacy of blocking MIF-CD74 as a therapeutic approach for meningioma treatment.

Our study emphasizes common manifestations of five pathological types of meningiomas and analyzed a public dataset to support our observations. However, it requires further efforts to verify these findings in other subtypes of meningiomas. Furthermore, we underscored the heterogeneity of neoplastic cells and macrophages in meningiomas which should be further validated in a larger sample size. We must emphasize that although pathway enrichment and cell fate analysis suggest two states of macrophages, further analysis with more samples will be required in the future to exclude the influence of patient age, individual immune status, environmental factors, and other contributions to these two states. Validation of the activation status of relevant pathways and the true existence of these two states will be carried out through experiments at the protein and cellular levels. As our current research findings have only been validated in a limited number of single-cell samples from meningiomas, we emphasize the necessity for future validation in larger cohorts to enhance the reliability and generalizability of the results. Subsequent investigations may also consider employing spatial transcriptomics techniques to exclude false positive cell communication results that may arise due to distant cell proximity, thereby providing a more accurate assessment of heterogeneity [50].

## Conclusions

In summary, through single-cell transcriptomic analysis of meningioma samples, we identified heterogeneity among tumor cells of different pathological subtypes of meningioma, a 'pro-tumorigenic' state of macrophages, and the ubiquitous presence of *MIF*-*CD74* interaction. We propose that blocking *CD74*-positive macrophages from receiving *MIF* signals and inhibiting their progression to a pro-tumorigenic state may be a key therapeutic strategy for treating different types of meningiomas.

### Supplementary Information

**Additional file 1: Figure S1.** Marker genes for cell type identification. **A** Heatmap of top 10 marker genes for 6 cell types.**B** Umap plot showing expression of *CLU*, *CD74*, *CD3D*, *CD34*, *ACTA2*, and *MAG* in all cells. **C** Violin plots showing expression of *CLU*, *CD74*, *CD3D*, *CD34*, *ACTA2*, and *MAG* in all cells.

**Additional file 2: Figure S2.** Integration of public dataset samples and cell type identification. **A** UMAP plot of single cells after integration of samples from public datasets, clustered into 23 clusters. Each point depicts a single cell. **B** and **C** Expression of marker genes for the 6 identified cell types and for each individual cell type in our dataset: neoplastic cells (*CLU*, *PTN*, *LEPR*, and *SSTR2*); macrophages (*HLA-DRB5*, *CD74*, *MS4A6A*, and *LYZ*); T cells (*CD3D*, *CD3E*, *CD3G*, and *CD52*); endothelial cells (*CD34*, *VWF*, *CCL14*, and* PLVAP*); fibroblasts (ACTA2 and *RGS5*); and oligodendrocytes (*CNP*, *MAG*, *KLK6*, and *OLIG2*). Scaled color bar represents average expression, size of the point represents percent expressed. **D** Proportions of the 6 identified cell types in each tissue. **E** Umap plot showing expression of *CLU*, *CD74*, *CD3D*, *CD34*, *ACTA2*, and *MAG* in all cells from the public dataset. **F** Violin plots showing expression of *CLU*, *CD74*, *CD3D*, *CD34*, *ACTA2*, and *MAG* in the 6 identified cell types from the public dataset.

**Additional file 3: Figure S3.** Neoplastic cell subclustering, pseudotime analysis, and WGCNA in a public dataset. **A** Marker genes of different subclusters of neoplastic cells in a public dataset. Scaled color bar = average expression, Size of the point = percent expressed.**B** Violin plots of *CD44* and *CD133* expression in neoplastic cells of a public dataset. **C** GO and KEGG enrichment analysis results (Top 5, p adjusted <0.05) of marker genes in cluster 12 of neoplastic cells in a public dataset. **D** Pseudotime analysis results of MSC6 neoplastic cells. The evolution of pseudotime relationship is shown on the left (scaled color bar = pseudotime). The distribution of neoplastic cell subclusters on the pseudotime trajectory is shown in the middle. The distribution of cluster 12 neoplastic cells on the pseudotime trajectory is shown on the right. Each cell in the branched pseudotime trajectory was colored by its pseudotime value and its Seurat clusters. **E** Expression of genes in 10 modules in different subclusters of neoplastic cells. Scaled color bar = average expression, Size of the point = percent expressed. **F** WGCNA analysis of neoplastic cells in a public dataset, resulting in 10 modules (GSE-NEO1-GSE-NEO10) of genes represented by different colors, except for the gray module. **G** Expression of top 50 genes in each module in the UMAP of neoplastic cells in a public dataset.

**Additional file 4: Figure S4.** Neoplastic cell gene regulatory network. **A.** Heatmap of the average regulon activity score for each neoplastic cell subpopulation. **B.** The regulon specificity score heat map of 99 regulon in each cell.

**Additional file 5: Figure S5.**Expression of classical marker genes for M1 and M2 macrophages in the macrophage UMAP plot.**A** Expression of classical marker genes for M1 macrophages **B** Expression of classical marker genes for M2 macrophages.

**Additional file 6 Figure S6.** Subclustering and pseudotime analysis of macrophages in a public dataset. **A** The subclustering of macrophages in a public dataset into 10 subclusters. Each point represents a cell, color-coded by their associated cluster. **B** The separation of macrophage clustering in the public dataset, showing similarity in macrophage distribution between tissues from the same sample (e.g. MSC5 and MSC5_BTI; MSC6 and MSC6_BTI). **C** The marker gene expression of macrophage subclusters in the public dataset. Scaled color bar represents the average expression, and size of the point represents the percentage expressed. **D** Pseudotime analysis of macrophages in the public dataset. The pseudotime trajectory is shown on the left with color-coded pseudotime values, and the distribution of macrophages in pseudotime states is shown on the right. Each cell in the branched pseudotime trajectory was colored by its pseudotime value and its states. **E.** Distribution of early pseudotime macrophages in the public dataset, mainly located in state1 and state5.**F** GO enrichment analysis results of highly expressed genes in early pseudotime macrophages, showing a grouping network.**G** Heatmap of highly expressed genes in different cell fates during pseudotime analysis. Scaled color bar represents the average expression. Highly expressed genes in the early pseudotime period are concentrated on the left, while highly expressed genes in the late pseudotime period are concentrated on the right. **H** Distribution of late pseudotime macrophages in the public dataset, mainly located in state3 and state4. **I** GO enrichment analysis results of highly expressed genes in late pseudotime macrophages, showing a grouping network.

**Additional file 7: Figure S7.** Expression of marker genes for M1 and M2 macrophages in the public dataset. **A** Expression of classical marker genes for M1 macrophages in macrophages from the public dataset. **B** Expression of classical marker genes for M2 macrophages in macrophages from the public dataset.

**Additional file 8: Figure S8.** Communication probabilities of all ligand-receptor pairs in cell communication results. p-value <0.01, Scaled color bar = communication probability.

**Additional file 9:  Figure S9.** Intercellular communication analysis in meningiomas in the public dataset. **A** Number of interactions network among cells in the public dataset. The thickness of the lines represents the number of interactions. **B** Interaction weight network among cells in the public dataset. The thickness of the lines represents the interaction weight. **C** Chord plot showing the inferred intercellular communication network of *MIF* signaling in the public dataset. **D** Heatmap of communication probability in *MIF* signaling in the public dataset, scaled color bar = Communication Probability. **E** Violin plots of ligand and receptor genes (*MIF*, *CD74*, *CXCR4*, and *CD44*) expression in cells in the MIF signaling in the public dataset. **F** Ligand-receptor pairs included in the *MIF* signaling and their relative contribution in the public dataset. **G** UMAP plot showing cells expressing *MIF*, *CD74*, *CXCR4*, and *CD44* colored in the public dataset.

**Additional file 10: Figure S10.** Multiple immunofluorescence staining of different pathological types of meningioma tissues. **A** Multiple immunofluorescence staining of atypical, transitional,anaplastic and clear cell meningioma. The staining includes *MIF* (green fluorescence), *CD74* (red fluorescence), and DAPI (blue fluorescence). Scale bar, 50 μm

**Additional file 11 Table S1.** The quantity of six distinct cellular types present in the specimen.

**Additional file 12: Table S2.** Gene list of WGCNA modules in neoplastic cells.

**Additional file 13:** **Table S3.** GO enrichment analysis results for WGCNA modules in neoplastic cells.

**Additional file 14: Table S4.** Gene lists for WGCNA Modules in neoplastic cells from public dataset gse183655.

**Additional file 15: Table S5.** GO enrichment analysis results for wgcna modules in neoplastic cells from public dataset GSE183655.

**Additional file 16: Table S6.** State of each macrophage in pseudotime analysis.

**Additional file 17: Table S7.** Highly expressed genes in macrophages across different cell fates.

**Additional file 18: Table S8.** Function of 33 Cell Communication Signals.

## Data Availability

The datasets supporting the conclusions of this article are available in the Genome Sequence Archive [14]. Each sample's files are hyperlinked to the dataset on https://bigd.big.ac.cn/gsa-human/browse/HRA004857.

## Abbreviations

BTI: Brain-tumor interface
BEAM: Branched expression analysis modeling
CNS: Central nervous system
GO: Gene ontology
KEGG: Kyoto Encyclopedia of Genes and Genomes
MIF: Macrophage migration inhibitory factor
scRNA-seq: Single-cell RNA sequencing
TME: Tumor microenvironment
TSCs: Tumor stem cells
UMAP: Uniform manifold approximation and projection
WGCNA: Weighted gene co-expression network analysis
