# Supplementary figures and images for: Decoding meningioma heterogeneity and neoplastic cell—macrophage interaction through single-cell transcriptome profiling across pathological grades
Source: J Transl Med. 2023 Oct 25;21:751. doi: 10.1186/s12967-023-04445-4 (PMC10599053; doi:10.1186/s12967-023-04445-4)

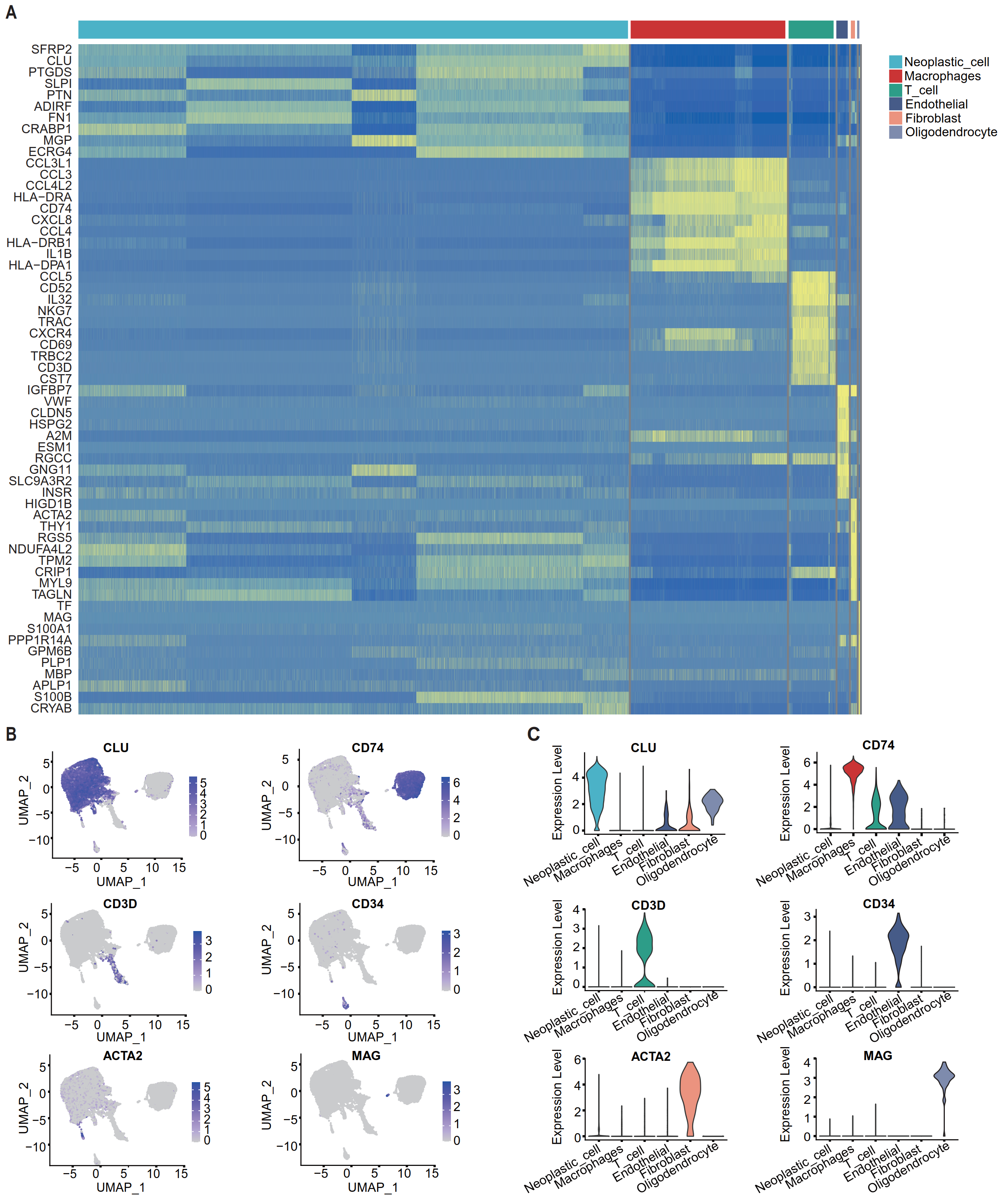

Supplement: Supplementary file 1 — Additional file 1: Figure S1. Marker genes for cell type identification. A Heatmap of top 10 marker genes for 6 cell types.B Umap plot showing expression of CLU, CD74, CD3D, CD34, ACTA2, and MAG in all cells. C Violin plots showing expression of CLU, CD74, CD3D, CD34, ACTA2, and MAG in all cells. [file 12967_2023_4445_MOESM1_ESM.tif]

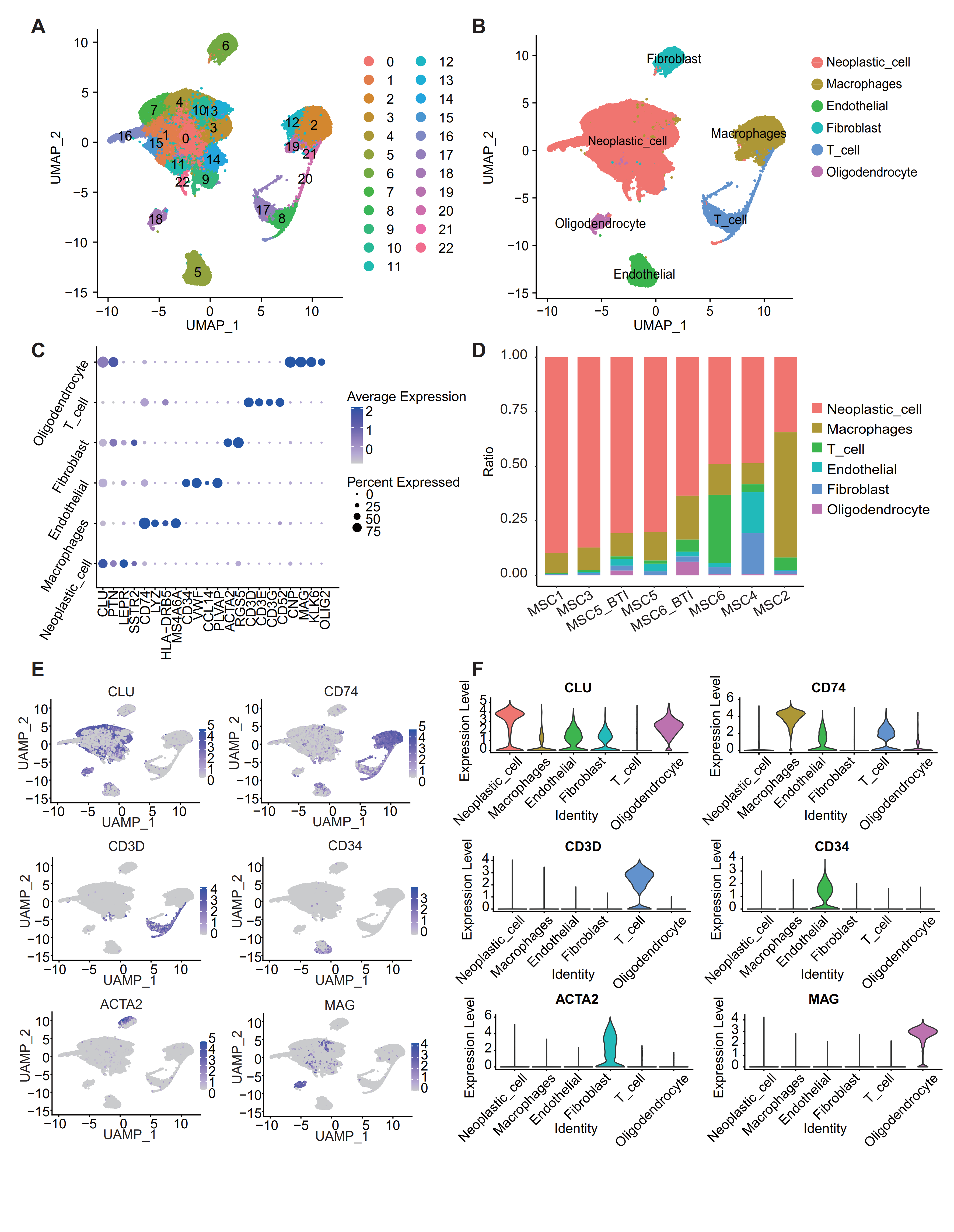

Supplement: Supplementary file 2 — Additional file 2: Figure S2. Integration of public dataset samples and cell type identification. A UMAP plot of single cells after integration of samples from public datasets, clustered into 23 clusters. Each point depicts a single cell. B and C Expression of marker genes for the 6 identified cell types and for each individual cell type in our dataset: neoplastic cells (CLU, PTN, LEPR, and SSTR2); macrophages (HLA-DRB5, CD74, MS4A6A, and LYZ); T cells (CD3D, CD3E, CD3G, and CD52); endothelial cells (CD34, VWF, CCL14, and PLVAP); fibroblasts (ACTA2 and RGS5); and oligodendrocytes (CNP, MAG, KLK6, and OLIG2). Scaled color bar represents average expression, size of the point represents percent expressed. D Proportions of the 6 identified cell types in each tissue. E Umap plot showing expression of CLU, CD74, CD3D, CD34, ACTA2, and MAG in all cells from the public dataset. F Violin plots showing expression of CLU, CD74, CD3D, CD34, ACTA2, and MAG in the 6 identified cell types from the public dataset. [file 12967_2023_4445_MOESM2_ESM.tif]

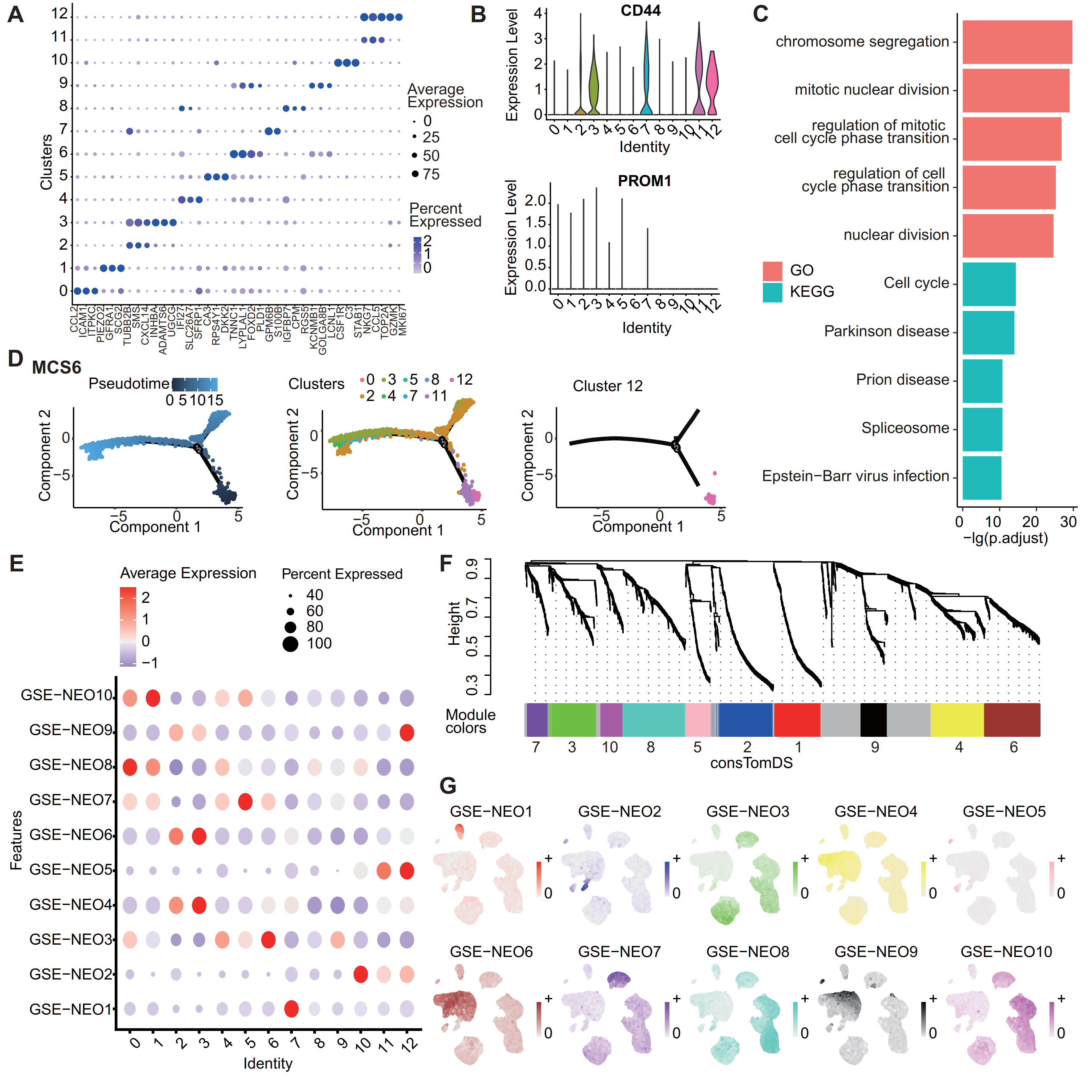

Supplement: Supplementary file 3 — Additional file 3: Figure S3. Neoplastic cell subclustering, pseudotime analysis, and WGCNA in a public dataset. A Marker genes of different subclusters of neoplastic cells in a public dataset. Scaled color bar = average expression, Size of the point = percent expressed.B Violin plots of CD44 and CD133 expression in neoplastic cells of a public dataset. C GO and KEGG enrichment analysis results (Top 5, p adjusted <0.05) of marker genes in cluster 12 of neoplastic cells in a public dataset. D Pseudotime analysis results of MSC6 neoplastic cells. The evolution of pseudotime relationship is shown on the left (scaled color bar = pseudotime). The distribution of neoplastic cell subclusters on the pseudotime trajectory is shown in the middle. The distribution of cluster 12 neoplastic cells on the pseudotime trajectory is shown on the right. Each cell in the branched pseudotime trajectory was colored by its pseudotime value and its Seurat clusters. E Expression of genes in 10 modules in different subclusters of neoplastic cells. Scaled color bar = average expression, Size of the point = percent expressed. F WGCNA analysis of neoplastic cells in a public dataset, resulting in 10 modules (GSE-NEO1-GSE-NEO10) of genes represented by different colors, except for the gray module. G Expression of top 50 genes in each module in the UMAP of neoplastic cells in a public dataset. [file 12967_2023_4445_MOESM3_ESM.tif]

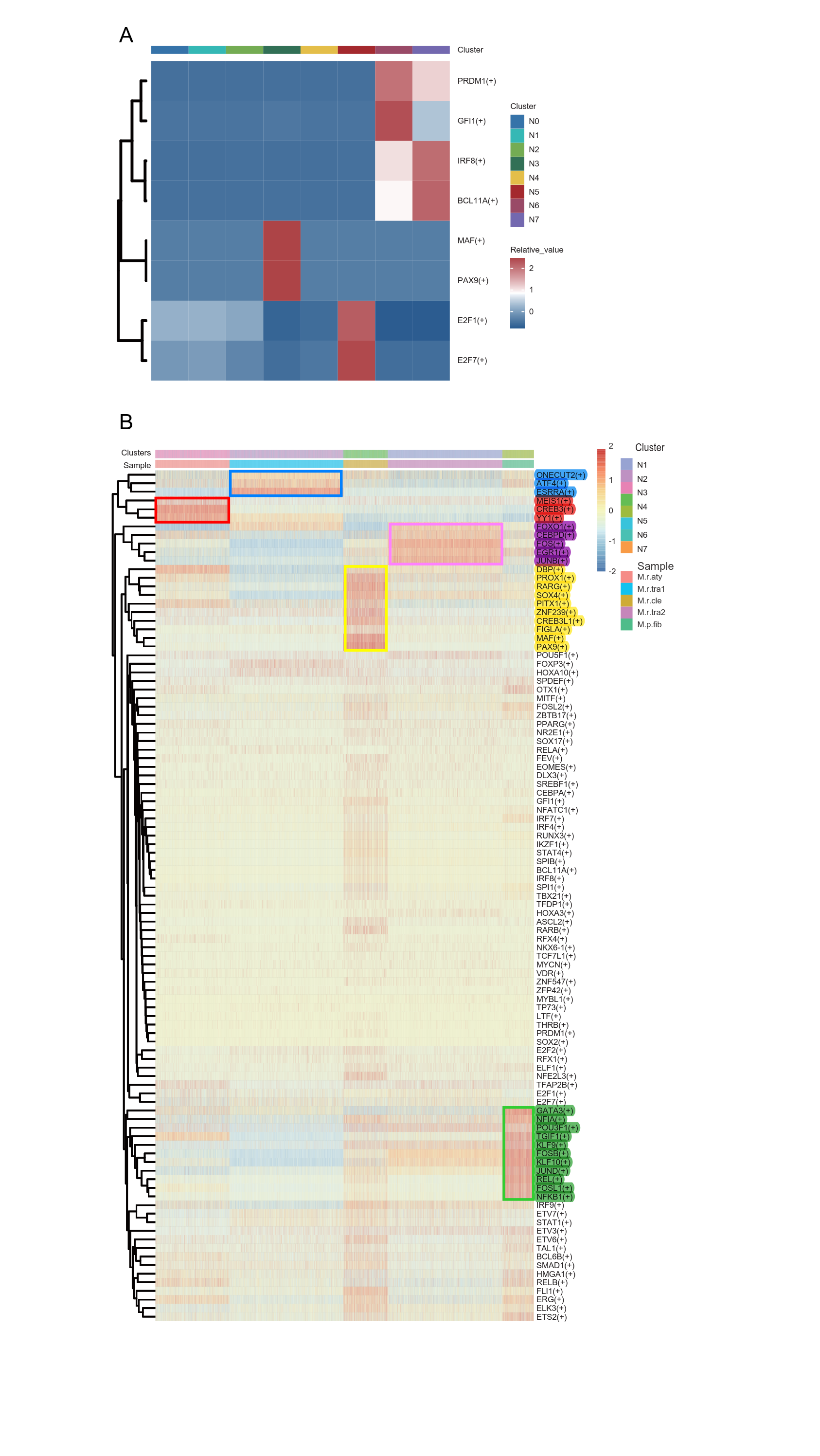

Supplement: Supplementary file 4 — Additional file 4: Figure S4. Neoplastic cell gene regulatory network. A. Heatmap of the average regulon activity score for each neoplastic cell subpopulation. B. The regulon specificity score heat map of 99 regulon in each cell. [file 12967_2023_4445_MOESM4_ESM.tif]

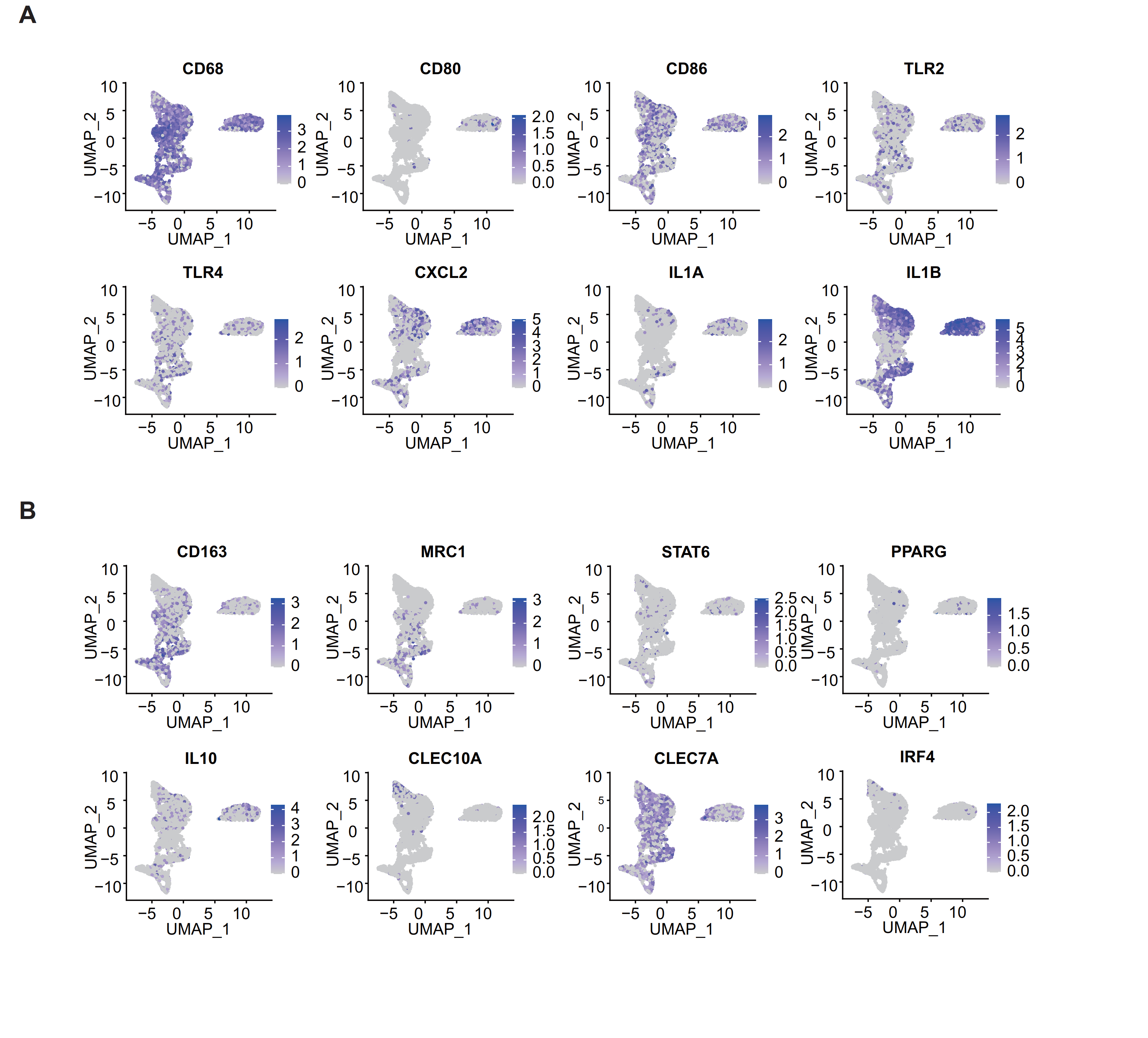

Supplement: Supplementary file 5 — Additional file 5: Figure S5.Expression of classical marker genes for M1 and M2 macrophages in the macrophage UMAP plot.A Expression of classical marker genes for M1 macrophages B Expression of classical marker genes for M2 macrophages. [file 12967_2023_4445_MOESM5_ESM.tif]

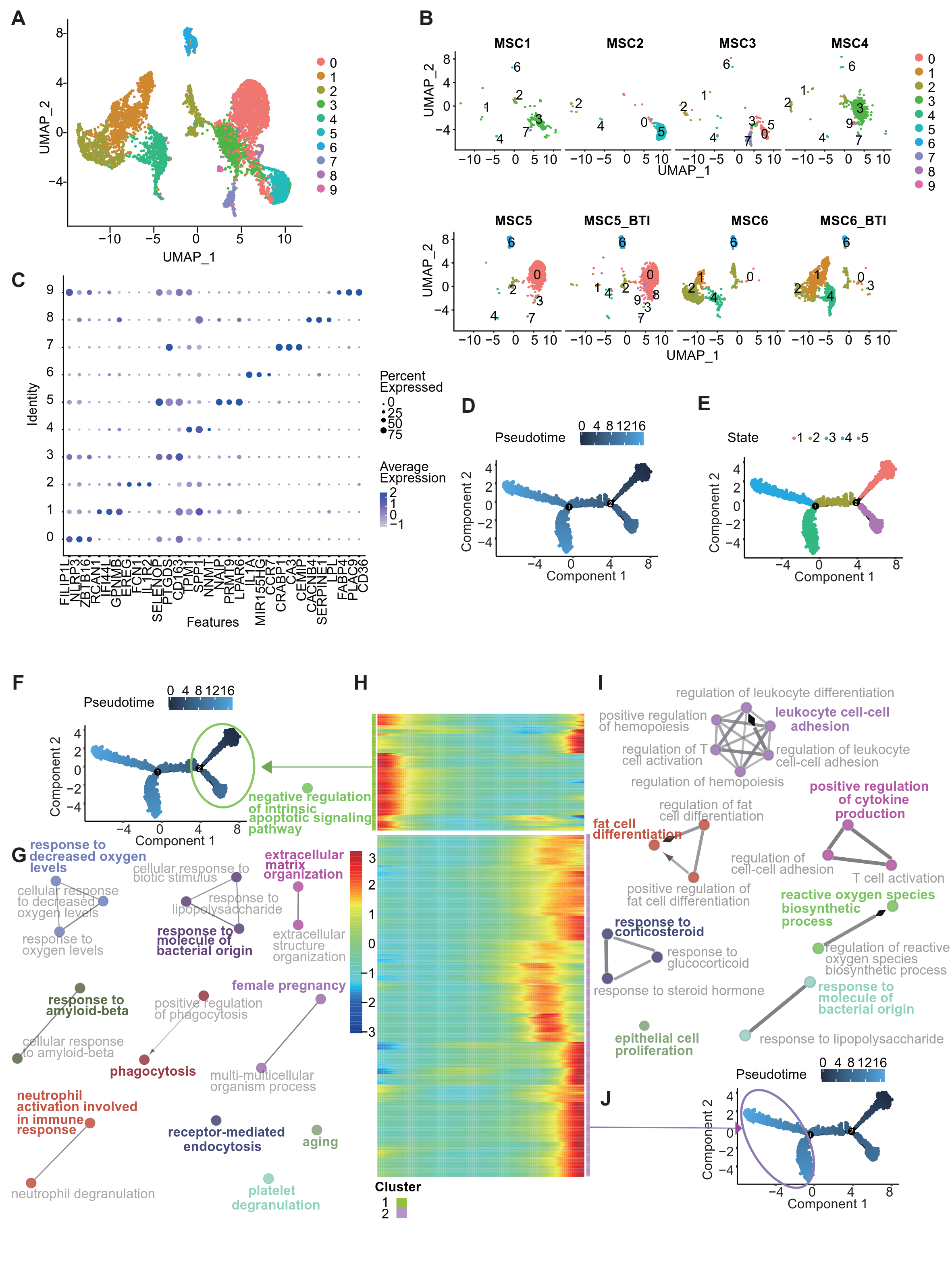

Supplement: Supplementary file 6 — Additional file 6 Figure S6. Subclustering and pseudotime analysis of macrophages in a public dataset. A The subclustering of macrophages in a public dataset into 10 subclusters. Each point represents a cell, color-coded by their associated cluster. B The separation of macrophage clustering in the public dataset, showing similarity in macrophage distribution between tissues from the same sample (e.g. MSC5 and MSC5_BTI; MSC6 and MSC6_BTI). C The marker gene expression of macrophage subclusters in the public dataset. Scaled color bar represents the average expression, and size of the point represents the percentage expressed. D Pseudotime analysis of macrophages in the public dataset. The pseudotime trajectory is shown on the left with color-coded pseudotime values, and the distribution of macrophages in pseudotime states is shown on the right. Each cell in the branched pseudotime trajectory was colored by its pseudotime value and its states. E. Distribution of early pseudotime macrophages in the public dataset, mainly located in state1 and state5.F GO enrichment analysis results of highly expressed genes in early pseudotime macrophages, showing a grouping network.G Heatmap of highly expressed genes in different cell fates during pseudotime analysis. Scaled color bar represents the average expression. Highly expressed genes in the early pseudotime period are concentrated on the left, while highly expressed genes in the late pseudotime period are concentrated on the right. H Distribution of late pseudotime macrophages in the public dataset, mainly located in state3 and state4. I GO enrichment analysis results of highly expressed genes in late pseudotime macrophages, showing a grouping network. [file 12967_2023_4445_MOESM6_ESM.tif]

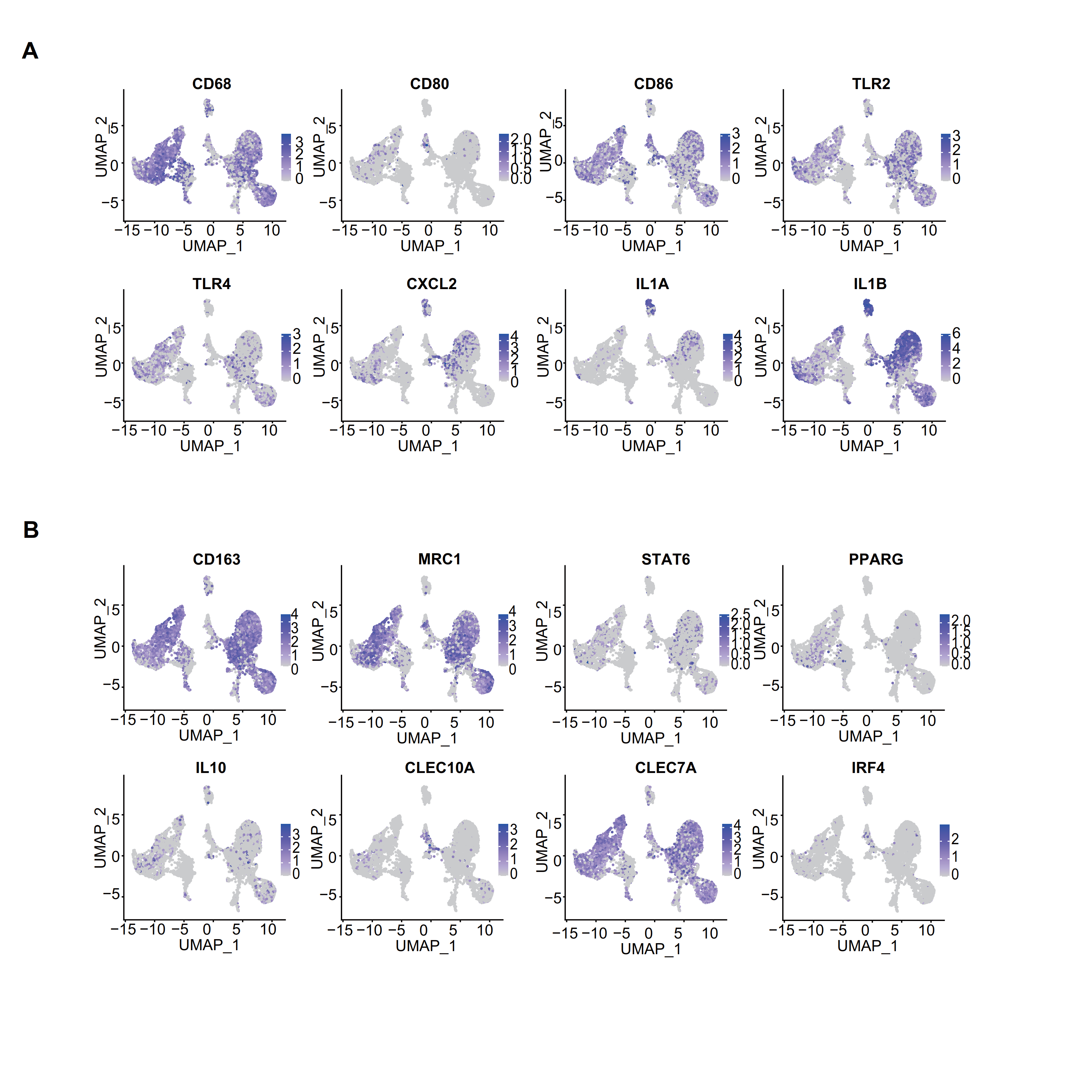

Supplement: Supplementary file 7 — Additional file 7: Figure S7. Expression of marker genes for M1 and M2 macrophages in the public dataset. A Expression of classical marker genes for M1 macrophages in macrophages from the public dataset. B Expression of classical marker genes for M2 macrophages in macrophages from the public dataset. [file 12967_2023_4445_MOESM7_ESM.tif]

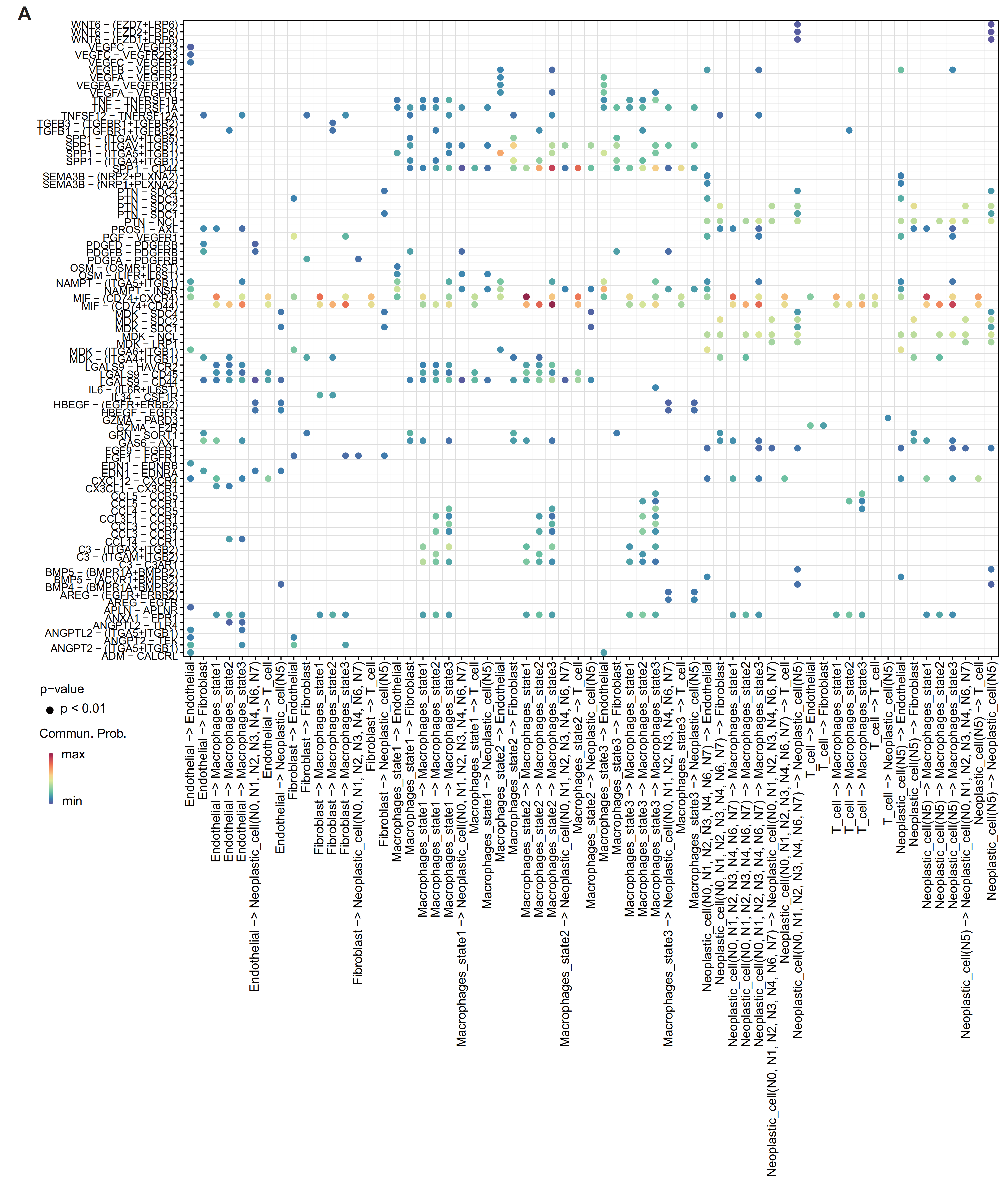

Supplement: Supplementary file 8 — Additional file 8: Figure S8. Communication probabilities of all ligand-receptor pairs in cell communication results. p-value <0.01, Scaled color bar = communication probability. [file 12967_2023_4445_MOESM8_ESM.tif]

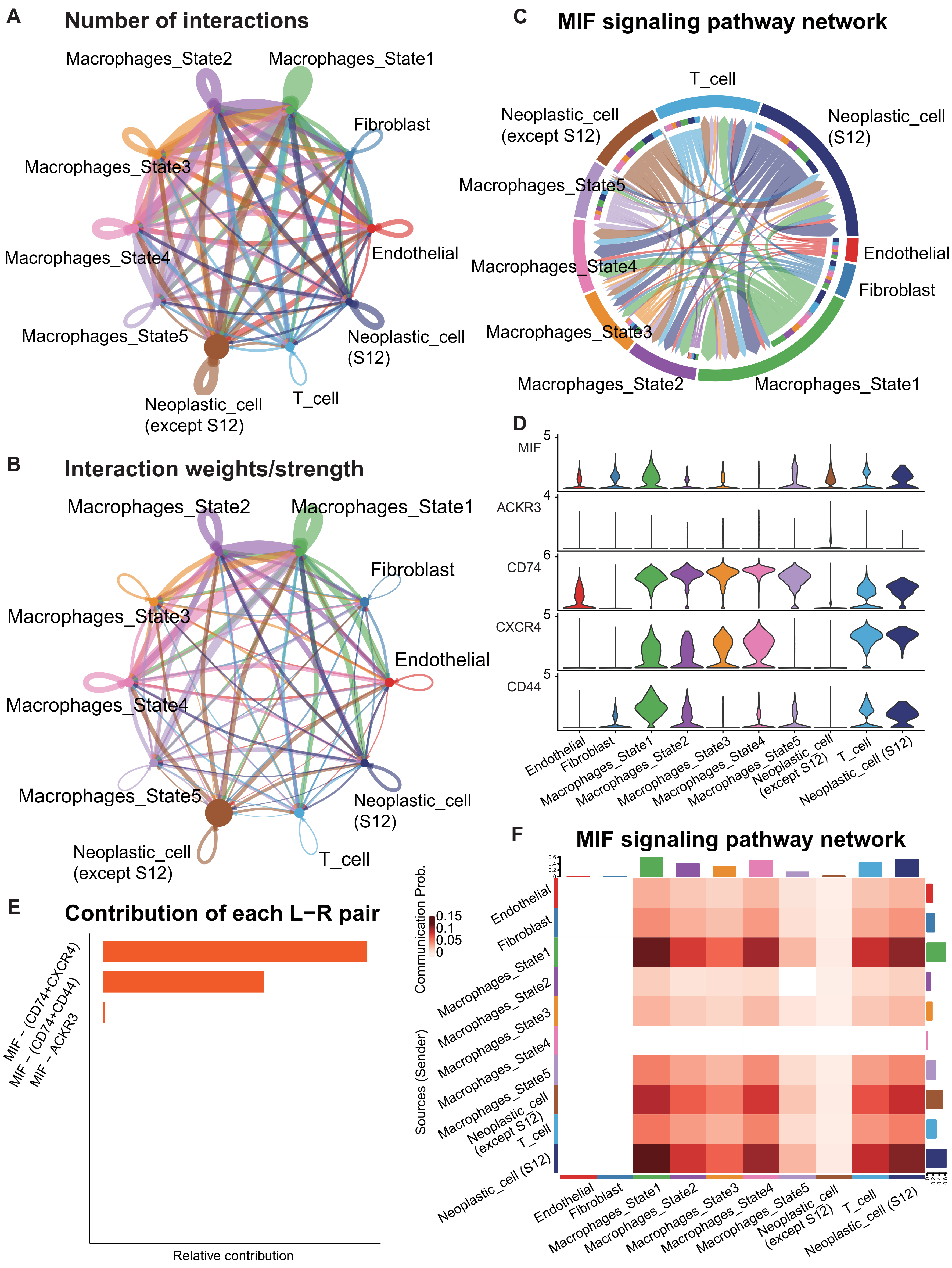

Supplement: Supplementary file 9 — Additional file 9: Figure S9. Intercellular communication analysis in meningiomas in the public dataset. A Number of interactions network among cells in the public dataset. The thickness of the lines represents the number of interactions. B Interaction weight network among cells in the public dataset. The thickness of the lines represents the interaction weight. C Chord plot showing the inferred intercellular communication network of MIF signaling in the public dataset. D Heatmap of communication probability in MIF signaling in the public dataset, scaled color bar = Communication Probability. E Violin plots of ligand and receptor genes (MIF, CD74, CXCR4, and CD44) expression in cells in the MIF signaling in the public dataset. F Ligand-receptor pairs included in the MIF signaling and their relative contribution in the public dataset. G UMAP plot showing cells expressing MIF, CD74, CXCR4, and CD44 colored in the public dataset. [file 12967_2023_4445_MOESM9_ESM.tif]

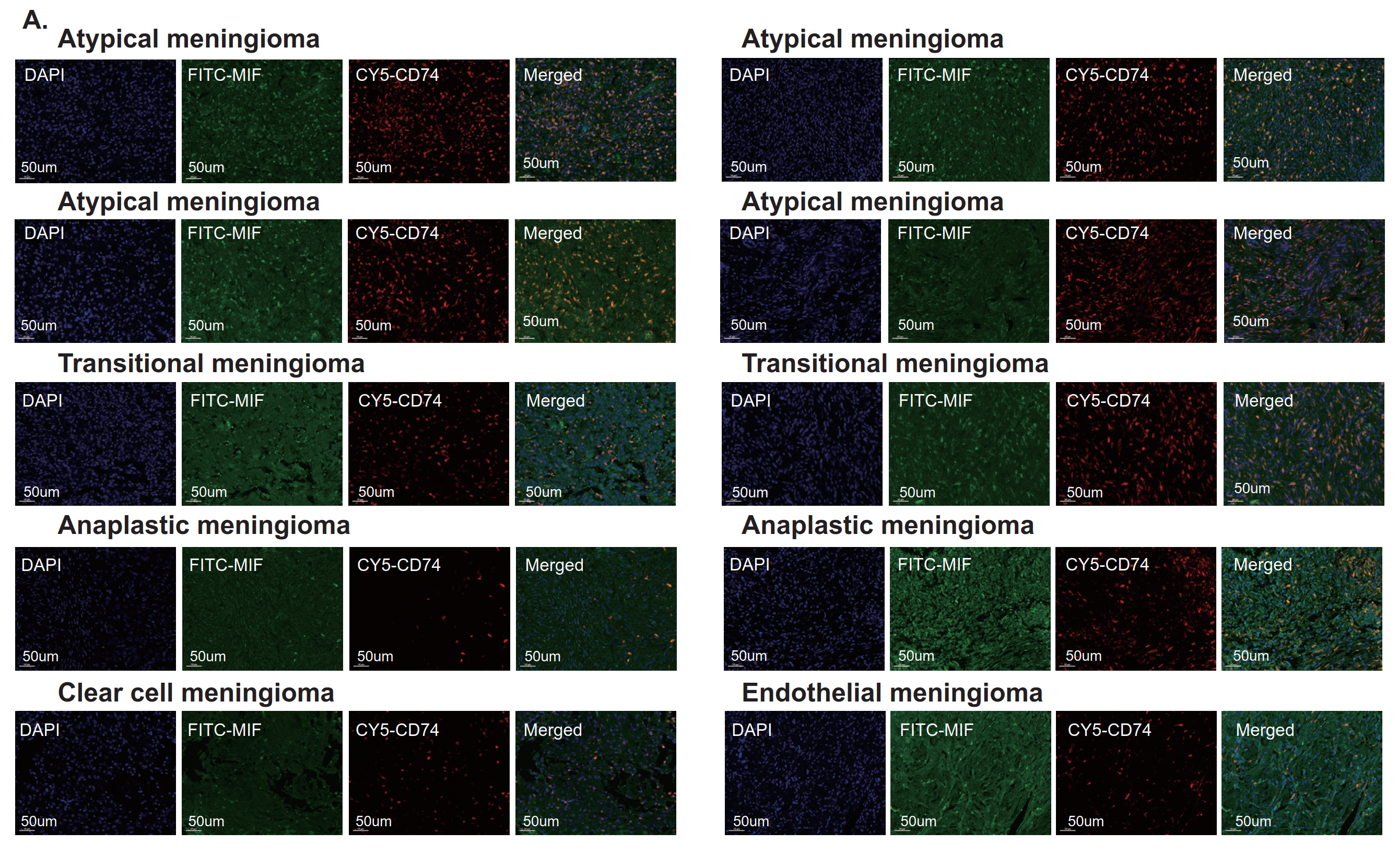

Supplement: Supplementary file 10 — Additional file 10: Figure S10. Multiple immunofluorescence staining of different pathological types of meningioma tissues. A Multiple immunofluorescence staining of atypical, transitional,anaplastic and clear cell meningioma. The staining includes MIF (green fluorescence), CD74 (red fluorescence), and DAPI (blue fluorescence). Scale bar, 50 μm [file 12967_2023_4445_MOESM10_ESM.tif]
